# Effect of Micellar Morphology on the Temperature-Induced Structural Evolution of ABC Polypeptoid Triblock Terpolymers into Two-Compartment Hydrogel Network

**DOI:** 10.1021/acs.macromol.4c00162

**Published:** 2024-06-28

**Authors:** Naisheng Jiang, Tianyi Yu, Meng Zhang, Bailee N. Barrett, Haofeng Sun, Jun Wang, Ying Luo, Garrett L. Sternhagen, Sunting Xuan, Guangcui Yuan, Elizabeth G. Kelley, Shuo Qian, Peter V. Bonnesen, Kunlun Hong, Dongcui Li, Donghui Zhang

**Affiliations:** † Key Laboratory of Advanced Materials and Devices for Post-Moore Chips, Ministry of Education, School of Materials Science and Engineering, University of Science and Technology Beijing, Beijing 100083, China; ‡ Department of Chemistry and Macromolecular Studies Group, Louisiana State University, Baton Rouge, Louisiana 70803, United States; § NIST Center for Neutron Research, National Institute of Standards and Technology, Gaithersburg, Maryland 20899, United States; ∥ Neutron Scattering Division and Second Target Station, Oak Ridge National Laboratory, Oak Ridge, Tennessee 37831, United States; ⊥ Center for Nanophase Materials Sciences, Oak Ridge National Laboratory, Oak Ridge, Tennessee 37831, United States; # Hua An Tang Biotech Group Co., Ltd., Guangzhou 511434, China

## Abstract

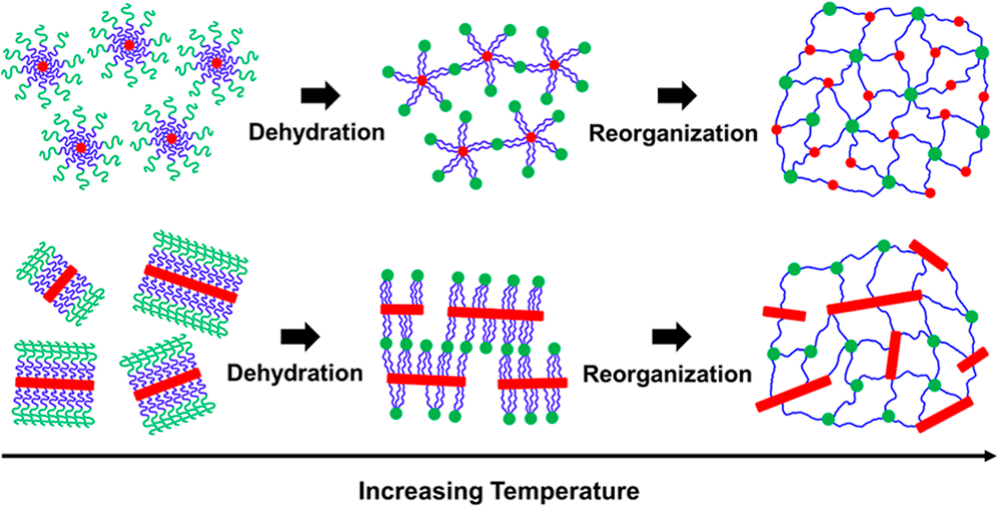

We investigated the temperature-dependent structural evolution of thermoreversible triblock terpolypeptoid hydrogels, namely poly(*N*-allyl glycine)-*b*-poly(*N*-methyl glycine)-*b*-poly(*N*-decyl glycine) (AMD), using small-angle neutron scattering (SANS) with contrast matching in conjunction with X-ray scattering and cryogenic transmission electron microscopy (cryo-TEM) techniques. At room temperature, A_100_M_101_D_10_ triblock terpolypeptoids self-assemble into core–corona-type spherical micelles in aqueous solution. Upon heating above the critical gelation temperature (*T*_gel_), SANS analysis revealed the formation of a two-compartment hydrogel network comprising distinct micellar cores composed of dehydrated A blocks and hydrophobic D blocks. At *T* ≳ *T*_gel_, the temperature-dependent dehydration of A block further leads to the gradual rearrangement of both A and D domains, forming well-ordered micellar network at higher temperatures. For AMD polymers with either longer D block or shorter A block, such as A_101_M_111_D_21_ and A_43_M_92_D_9_, elongated nonspherical micelles with a crystalline D core were observed at *T* < *T*_gel_. Although these enlarged crystalline micelles still undergo a sharp sol-to-gel transition upon heating, the higher aggregation number of chains results in the immediate association of the micelles into ordered aggregates at the initial stage, followed by a disruption of the spatial ordering as the temperature further increases. On the other hand, fiber-like structures were also observed for AMD with longer A block, such as A_153_M_127_D_10_, due to the crystallization of A domains. This also influences the assembly pathway of the two-compartment network. Our findings emphasize the critical impact of initial micellar morphology on the structural evolution of AMD hydrogels during the sol-to-gel transition, providing valuable insights for the rational design of thermoresponsive hydrogels with tunable network structures at the nanometer scale.

## Introduction

Stimuli-responsive hydrogels composed of cross-linked polymeric networks capable of reversibly changing their structure and mechanical properties in response to external stimuli hold great promise for applications in drug delivery, cell therapy, and tissue engineering.^1^ Among them, thermoreversible and injectable hydrogels that undergo a sol-to-gel transition around physiological temperature (i.e., ∼37 °C) are particularly appealing due to their minimally invasive nature for in vivo use.^2−5^ While hydrogels derived from natural biopolymers such as collagen, alginate, and hyaluronic acid have found extensive applications, they are accompanied by inherent limitations in terms of molecular tunability, batch-to-batch variability, and potential immunological responses. To overcome these challenges, considerable advances have been made in the development of synthetic polymer-based hydrogels.^6,7^ One notable advantage of synthetic polymers lies in their ability to fine-tune the multiscale structure, thermal sensitivity, reversibility, cytocompatibility, degradability, and mechanical properties of the hydrogels through molecular design.^6,7^

Distinguished by the nature of their network junctions or cross-links, stimuli-responsive polymer-based hydrogels can be categorized into two main types: chemically cross-linked and physically cross-linked hydrogels. Chemically cross-linked hydrogels involve the formation of covalent linkages between polymer segments under external stimuli, while physically cross-linked hydrogels rely on noncovalent interactions, such as hydrogen bonding, hydrophobic interactions, ionic interactions, or topological entanglements, to create temporary and reversible cross-links.^8^ While chemically cross-linked hydrogels are well-known for their remarkable mechanical properties, physically cross-linked hydrogels can be formed without the need for harsh cross-linking conditions such as UV-radiation or the addition of cross-linking agents, thereby minimizing the potential for tissue damage upon hydrogel injection in biomedical uses.^9,10^ Additionally, physically cross-linked hydrogels demonstrate high gelation reversibility accompanied by conformational changes of polymer chains, allowing them to undergo repeated cycles of gelation and solvation.^2,7^ These advantages make physically cross-linked hydrogels particularly suitable for various biomedical applications where controlled and reversible gelation is desirable. However, it is worth noting that physically cross-linked hydrogels can be susceptible to syneresis, leading to shrinkage or collapse over time due to the gradual release of water from the gel network.^7,11^ In this regard, chemically cross-linked hydrogels tend to exhibit greater resistance to syneresis, providing enhanced long-term stability and structural integrity.

One effective approach to exploit the benefits of thermoresponsive physically cross-linked hydrogels is through the utilization of amphiphilic multiblock copolymers. Inherently, these polymers have the capability to self-assemble into physically cross-linked networks in aqueous solutions when exposed to certain temperatures. ABA triblock copolymers, which consist of a hydrophilic B midblock with hydrophobic A blocks on both ends, have traditionally been a focus for hydrogel formation.^12−18^ The aggregation of hydrophobic segments driven by hydrophobic interactions plays a crucial role in generating cross-linking sites within the polymer network, while the hydrophilic block ensures water penetration and retention within the hydrogel. To achieve thermoresponsive behavior, hydrophobic blocks with a lower critical solution temperature (LCST) such as poly(*N*-isopropylacrylamide) (PNIPAm) are commonly employed, as these blocks will undergo a phase transition from well-hydrated to dehydrated states upon heating. However, ABA triblock copolymers often face limitations in terms of gelation efficiency, such as requiring high polymer concentrations and exhibiting broad sol–gel transitions.^19^ These challenges arise from the presence of network defects, such as looped chains and dangling ends, which outweigh the contribution of bridging chains responsible for network elasticity.^20,21^ To overcome these limitations, the introduction of ABC triblock terpolymers with mutually immiscible hydrophobic A and C blocks has been proposed for designing thermoresponsive hydrogels.^19,21−24^ For example, Lodge and co-workers explored the thermoresponsive gelation behavior of poly(ethylene-*alt*-propylene)-*b*-poly(ethylene oxide)-*b*-poly(*N*-isopropylacrylamide) (PEP-*b*-PEO-*b*-PNIPAm) triblock copolymers using cryogenic electron microscopy (cryo-EM), small-angle neutron scattering (SANS) and rheological measurements.^19,22^ They showed that heating these ABC triblock terpolymers above the LCST of PNIPAm leads to the formation of a two-compartment hydrogel network, characterized by distinct PEP and PNIPAm domains bridged by PEO midblocks. This ABC terpolymer undergoes a sharper sol-gel transtion at lower critical gelation concentrations relative to the equivalent ABA triblock copolymers PNIPAm-*b*-PEO-*b*-PNIPAm.^19,22^

Polypeptoids, also known as *N*-substituted polyglycines, are a class of peptidomimetic polymers that have received considerable attention in recent years due to their potential in biomedical and biotechnological applications.^25−27^ In contrast to polypeptides, polypeptoids exhibit enhanced protease stability, good solubility and thermal processability, owing to the absence of hydrogen bonding and stereogenic centers along their N-substituted backbone.^25−27^ Moreover, they have demonstrated cytocompatibility with multiple cell lines^28,29^ and oxidative degradability under physiologically relevant conditions.^30^ In a recent study, Xuan et al. synthesized a series of ABC-type triblock terpolypeptoids, that is, poly(*N*-allyl glycine)-*b*-poly(*N*-methyl glycine)-*b*-poly(*N*-decyl glycine) (AMD), that exhibited thermo-reversible sol-to-gel transitions near the physiological temperature (∼37 °C) in aqueous solutions even at low concentrations ranging from 2.5 to 10 wt %.^31^ These injectable hydrogels showed minimal cytotoxicity toward human adipose-derived stem cells (hASCs) and efficiently encapsulated water-soluble enzymes while preserving their activity.^31^ The proposed gelation mechanism suggests that at lower temperatures, the triblock copolymers form core–shell-corona micelles, enabling injectability. Upon heating above the critical gelation temperature (*T*_gel_), the corona-forming poly(*N*-allyl glycine) (A) block undergoes dehydration due to LCST behavior,^31,32^ leading to the formation of a two-compartment micellar network. Anomalous fluctuations in storage and loss moduli of AMD hydrogels as a function of temperature were also observed at *T* > *T*_gel_,^31^ implying possible structural rearrangements within the hydrogel network. Yet, a comprehensive understanding of how temperature prompts structural evolution of the hydrogel network is elusive. Additionally, previous studies have indicated that certain polypeptoids, particularly those have long *n*-alkyl side chains like poly(*N*-decyl glycine), can crystallize even at low degrees of polymerization, leading to the emergence of anisotropic, nonspherical micelles in solution.^33−43^ However, how these crystallizable segments impact the early stage micellar morphology and the subsequent hydrogel formation is not fully understood.

In this contribution, we investigate the structural evolution and self-assembly of AMD triblock terpolypeptoid hydrogels during the sol-to-gel transition using a combination of contrast-matching SANS, solution small/wide-angle X-ray scattering (SAXS/WAXS) and cryogenic transmission electron microscopic (cryo-TEM) techniques. A series of poly(*N*-allyl glycine)-*b*-poly(*N*-methyl glycine)-*b*-poly(*N*-decyl glycine) (AMD) triblock terpolypeptoids, including partially deuterated poly(*N*-allyl glycine)-*b*-poly(*N*-methyl glycine)-*b*-poly(*N*-decyl-*d*_21_ glycine) (AMdD) counterparts, were synthesized through sequential benzyl amine-initiated ring-opening polymerization of the corresponding fully hydrogenated or partially deuterated N-substituted *N*-carboxyanhydrides (R-NCAs). We have found that in aqueous solution, triblock terpolypeptoids self-assemble into core–corona micelles, exhibiting either spherical or elongated nonspherical shapes, depending on the chain lengths of core-forming D block and thermoresponsive corona-forming A block. Upon heating above *T*_gel_, the micelles transform into a two-compartment hydrogel network with distinct hydrophobic domains composed of A and D blocks. SANS analysis allows us to elucidate the detailed structural change in the two-compartment network as a function of temperature. Intriguingly, the temperature-dependent structural evolution of the hydrogel network is significantly affected by the initial polymer micellar morphology in the solution, resulting in different spatial arrangement of the micellar domains. These findings highlight the intricate interplay between block composition, micellar shape, and structural evolution in ABC-type triblock hydrogels, which is important for the rational design of thermoresponsive polypeptoid hydrogels with tunable structures and properties.

## Experimental Section

### General Considerations

Deuterium oxide (D_2_O, 99.8 atom % D), deuterated dichloromethane (CD_2_Cl_2_, 99.8 atom % D), deuterated chloroform (CDCl_3_, 99.95 atom % D), and deuterated dimethyl sulfoxide-*d*_6_ (DMSO-*d*_6,_ 99.9 atom % D) were purchased from Cambridge Isotope Laboratories. All other chemicals and solvents were purchased from Sigma-Aldrich and used as received, unless stated otherwise. The solvents used for polymerization were purified using alumina columns under argon protection. Deionized water for hydrogel preparation was purified using a Barnstead Nanopure water purification system (Thermo Fisher Scientific, Waltham, MA, USA) with a resistivity of 18.2 MΩ·cm. ^1^H and ^2^H NMR spectra were collected by a Bruker AV-400 III spectrometer at 25 °C and analyzed using Topspin software. Chemical shifts (δ) are reported in parts per million (ppm) with reference to protio impurities of deuterated solvents. *N*-allyl glycine derived *N*-carboxyanhydride (Al-NCA) and *N*-methyl glycine derived *N*-carboxyanhydride (Me-NCA) and *N*-decyl glycine derived *N*-carboxyanhydride (De-NCA) monomers were synthesized according to the established procedures.^31,44^ For contrast matching SANS experiments, partially deuterated De-NCA monomer (De-*d*_21_-NCA) was also synthesized using *N*-decylamine-*d*_21_ provided by the Center for Nanophase Materials Sciences (CNMS) at Oak Ridge National Laboratory (ORNL), according to a published procedure.^31^ The detailed synthesis protocols and ^1^H NMR and ^2^H NMR spectra of these amines and NCA monomers are summarized in the Supporting Information (Schemes S1 and S2, Figure S1–S8).

### Synthesis of AMD and AMdD Triblock Terpolypeptoids

**Scheme 1:**
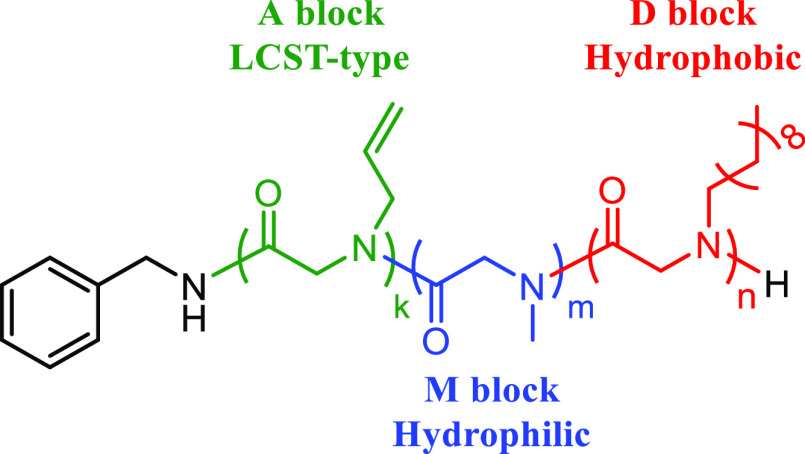
Chemical Structure of the AMD Triblock Terpolymer

Poly(*N*-allyl glycine)-*b*-poly(*N*-methyl glycine)-*b*-poly(*N*-decyl glycine) (AMD) (Scheme 1) were synthesized through sequential benzyl amine-initiated ring-opening polymerization of the corresponding N-substituted *N*-carboxyanhydrides (R-NCAs) (Scheme S3).^31^ To achieve neutron contrast matching, poly(*N*-allyl glycine)-*b*-poly(*N*-methyl glycine)-*b*-poly(*N*-decyl-*d*_21_ glycine) (AMdD) was also synthesized using De-*d*_21_-NCA following the same procedure. In brief, stock solutions of Al-NCA ([*M*_1_]_0_ = 0.4 M), Me-NCA ([*M*_2_]_0_ = 0.4 M), De-NCA ([*M*_3_]_0_ = 0.4 M), and De-*d*_21_-NCA ([*M*_3_]_0_ = 0.4 M) in anhydrous acetonitrile (CH_3_CN) were prepared in the glovebox. The polymerization of Al-NCA was conducted using a benzylamine initiator at 50 °C under a nitrogen atmosphere for 48 h to achieve complete conversion. Subsequently, Me-NCA and De-NCA or De-*d*_21_-NCA stock solutions were sequentially added at room temperature, resulting in complete conversion. The monomer-to-initiator ratio ([*M*_1_]_0_/[*M*_2_]_0_/[*M*_3_]_0_/[*I*]_0_) was controlled to yield triblock terpolypeptoids with targeted compositions of A_100_M_100_D_10_, A_100_M_100_D_20_, A_50_M_100_D_10_ and A_150_M_100_D_10_, respectively. Fourier-transform infrared (FT-IR) spectroscopy was used to confirm the conversion of each block by monitoring the disappearance of the −C=O peak at 1780 and 1740 cm^–1^. After completion of the polymerization, the solution was concentrated under vacuum, and the resulting polymer was precipitated by addition of hexane. The final polymer was isolated by centrifugation and drying under vacuum to afford a white powder (typical yield: ∼90%).

The composition of the AMD and AMdD triblock terpolymers was determined through end-group analysis by ^1^H NMR analysis (Table 1, Figures S9–S14). The number-averaged degree of polymerization (DP_*n*_) of the AMD polymers was determined by integrating the signals corresponding to the methyl protons in the D and M blocks at 0.91 and 3.0 ppm, as well as the terminal alkenyl protons in the A block at 5.8 ppm, relative to the integration of signals from the benzyl end-group at 7.3 ppm. For AMdD, the DP_*n*_ of the dD block was determined by ^1^H NMR analysis using the integration of the corresponding methylene protons on the polymer backbone at 3.7–4.2 ppm relative to that of the benzyl end-group protons at 7.3 ppm (Figure S13). The dispersity (*D̵*) of the polymers was determined using size-exclusion chromatography (SEC) coupled with a differential refractive index (dRI) detector in DMF with 0.1 M LiBr at 25 °C (Figure S15). All polymers exhibit a unimodal molecular weight distribution with *D̵* in the 1.08–1.18 range.

**Table 1:** Molecular Characteristics, Micellar Morphology, and Macroscopic Gelation Temperature of AMD Triblock Terpolypeptoids

polymer composition^a^	[*M*_1_]_0_/[*M*_2_]_0_/[*M*_3_]_0_/[*I*]_0_^b^	*M*_n_ (theor.) (kDa)^c^	*M*_n_ (NMR) (kDa)^d^	*M*_n_ (SEC) (kDa)^e^	*D̵*^e^	initial micellar morphology at 20 °C^f^	molecular ordering of core-forming D block^g^	*T*_gel_ (°C)^h^
A_100_M_101_D_10_	100/100/10/1	18.9	18.9	37.0	1.08	spherical	amorphous	33
A_95_M_99_dD_9_	100/100/10/1	18.9	18.2	43.1	1.15	spherical	amorphous	
A_101_M_107_D_21_	100/100/20/1	20.8	21.6	37.8	1.11	rod-like	liquid crystalline	33
A_96_M_105_dD_18_	100/100/20/1	20.8	20.7	45.3	1.18	rod-like	liquid crystalline	
A_43_M_92_D_9_	50/100/10/1	14.0	12.6	29.8	1.08	rod-like	liquid crystalline	
A_153_M_127_D_10_	150/100/10/1	27.2	25.8	36.2	1.08	fiber-like	amorphous	27

a The numbers in subscripts correspond to the DP_*n*_ of individual block determined by end-group analysis using ^1^H NMR spectroscopy in CD_2_Cl_2_. The molecular characteristics for A_43_M_92_D_9_ were reported previously.^31^

b Initial monomer to initiator ratio.

c Theoretical molecular weights were calculated from the initial monomer to initiator ratio.

d Determined by ^1^H NMR analysis.

e Determined by the SEC-DRI method using polystyrene standards (0.1 M LiBr/DMF, at 25 °C).

f Determined by cryo-TEM and SANS analysis.

g Determined by WAXS analysis.

h Macroscopic gelation temperatures of the 1 wt % aqueous solutions of the corresponding triblock terpolymers determined by rheological and UV–vis transmittance measurements according to previous study.^31^

### Preparation of the AMD Aqueous Solutions/Hydrogels

Aqueous solutions of AMD triblock terpolypeptoids at 5 wt % concentrations were prepared using D_2_O or nanopure deionized water (H_2_O) via the thin-film hydration method.^19,22,31^ In brief, the bulk polymers were initially dissolved in dichloromethane (DCM) in a glass vial to form a homogeneous solution with a concentration ≤5 mg/mL. The DCM solvent was then evaporated overnight under a nitrogen flow, leaving a thin film of polymers on the vial’s inner walls. The dried thin film was hydrated with D_2_O or H_2_O to achieve the targeted concentration. The solution was stirred at room temperature for at least 12 h under 300 rpm prior to further characterization. All AMD triblock terpolypeptoids used in this study exhibited thermoreversible gelation in D_2_O or H_2_O, as confirmed by rheological measurements and visual observation. When heated above 30–40 °C, the 5 wt % AMD solutions formed opaque gels, which returned to a free-flowing liquid state upon cooling to room temperature. For X-ray/neutron scattering and cryo-TEM measurements, the 1 wt % AMD solutions were prepared by direct dilution of the 5 wt % AMD solutions.

### Size-Exclusion Chromatography

SEC experiments were performed in DMF with 0.1 M LiBr at 25 °C with a flow rate of 0.5 mL/min. AMD or AMdD polymers were prepared into 5 mg/mL solution in SEC solvent and left to stand for overnight. The polymer solutions were filtered with 0.45 μm PTFE filters before injecting into the SEC system. SEC analysis was performed using an Agilent 1200 system equipped with three Phenomenex 5 μm, 300 mm × 7.8 mm columns, a Wyatt DAWN EOS multiangle light scattering (MALS) detector (GaAs 30 mW laser at λ = 690 nm) and Wyatt Optilab rEX differential refractive index (dRI) detector (Wyatt, Santa Barbara, CA). The data analysis was performed using Wyatt Astra V 5.3 software. The dispersity (*D̵*) values were determined using a calibration curve generated with polystyrene standards.

### Cryo-Transmission Electron Microscopy

Cryo-TEM imaging was performed using a FEI G2 F30 Tecnai TEM operated at 200 kV or a FEI Talos L120C TEM operated at 120 kV. A 5-μL droplet of the 1 wt % AMD solution was applied to a 200-mesh lacey carbon grid (Electron Microscopy Sciences) or a 300-mesh holey carbon gold grid (Beijing Zhongjingkeyi Technology) mounted on the FEI Vitrobot. Excess liquid was removed by blotting the grid with filter paper for 2–5 s, resulting in a thin sample film. The grid was then rapidly plunged into liquid ethane to vitrify the sample film. The vitrified sample was subsequently transferred to a single tilt cryo specimen holder for imaging. To capture the morphology at high temperature, the polymer solutions were heated to 40 °C for 10 min. A 5-μL droplet of the gel-like sample was then quickly transferred to a carbon grid mounted on the FEI Vitrobot, blotted for 2 s, and rapidly quenched in liquid ethane.

### Small-Angle Neutron Scattering

SANS data were collected at the National Institute of Standards and Technology Center for Neutron Research (NCNR) (Gaithersburg, MD) using two instruments: the NGB 30 m-SANS instrument and the NG3 vSANS instrument.^45^ The NGB 30 m-SANS instrument utilized two neutron wavelengths, λ = 6 Å and λ = 8.4 Å (Δλ/λ ∼ 0.14) with sample-to-detector distances of 13.2, 4.00 and 1.33 m, covering an effective *q*-range of ∼0.001–0.47 Å^–1^, where *q* is the magnitude of the scattering vector, which is described as *q* = 4π sin θ/λ, where θ is one-half of the scattering angle. The NG3 vSANS instrument employed a neutron wavelength of λ = 11 Å (Δλ/λ ∼ 0.12) with sample-to-detector distances of 4.5 and 18.5 m, yielding an effective *q*-range of ∼0.0013–0.48 Å^–1^. All samples were measured by using custom-made titanium cells with quartz windows and a path length of 2 mm, which were mounted on a temperature-controlled multiple position sample holder. The temperature of the sample holder was controlled by using a recirculation bath in a range of 15–60 °C with an accuracy better than 0.1 °C. To ensure sample equilibrium at the given temperatures, all samples were equilibrated for 30 min prior to each SANS measurement. A typical SANS data reduction protocol was used to correct for empty sample cell, background radiation, detector sensitivity, instrument dark current and sample transmission by using the Igor Pro 6.37 software (WaveMetrics, Inc., Lake Oswego, OR, USA) incorporated with data reduction macros.^46^

Additional SANS data were collected using the Bio-SANS instrument at the High Flux Isotope Reactor (HFIR), Oak Ridge National Laboratory (Oak Ridge, TN). The Bio-SANS instrument utilized two neutron wavelengths, λ = 6 Å and λ = 18 Å (Δλ/λ ∼ 0.15), with sample-to-detector distances of 15.5 and 1.1 m, covering an effective *q*-range of ∼0.001–0.8 Å^–1^. All samples were measured by using quartz banjo cells (Hellma USA, Plainview, NY) with a path length of 2 mm, which were mounted on a temperature-controlled multiple position sample holder. A typical SANS data reduction protocol was used to correct for instrument dark current, detector sensitivity, detector geometry, incident beam normalization and sample transmission by using the facility supplied data reduction software Mantid.^47^

Reduced two-dimensional (2D) SANS data were azimuthally averaged and merged from the two-detector setup to generate one-dimensional SANS intensity profiles, *I*(*q*) versus *q*, which were then scaled to an absolute cross section (units of cm^–1^). In this study, no attempt was made to subtract the incoherent scattering of the solvent (i.e., D_2_O or D_2_O/H_2_O mixture) from the SANS data due to the difficulty in accurately estimating it. Instead, the incoherent scattering of the solvent was included in a *q*-independent background term, along with the incoherent scattering from the hydrogen components in the sample, in the model fitting. The data were fitted using the SasView software package (version 5.0.6) available at https://www.sasview.org/. Instrumental smearing effects for SANS were included during SANS data fitting by convolving the scattering intensity with the SANS instrumental resolution function,^46^ which is incorporated in the SasView program.

### Solution X-Ray Scattering

Synchrotron-based small/wide-angle X-ray scattering (SAXS/WAXS) measurements were performed at the DND-CAT 5-ID-D beamline at the Advanced Photon Source (Argonne National Laboratory, Argonne, IL), using an X-ray wavelength of 1.37 Å (equivalent to an X-ray energy of 9.0 keV). Two wide-angle X-ray scattering (WAXS) 2D patterns along with a small-angle X-ray scattering (SAXS) 2D pattern were collected simultaneously on three Rayonix area CCD detectors at sample-to-detector distances of approximately 8.5, 1.0 and 0.2 m, respectively. This configuration covered an effective *q*-range of ∼0.0015–1.7 Å^–1^, which was calibrated using silver behenate. Samples were measured in a capillary flow cell at room temperature with a 1.5 mm nominal diameter quartz capillary (Charles Supper Company). The inner diameter of the capillary cell is estimated to be 1.47 mm from X-ray scans. The 2D scattering patterns were azimuthally isotropic and were azimuthally integrated around the beam center to produce one-dimensional *I*(*q*) versus *q* scattering profiles. The final X-ray curves for each sample were obtained by averaging two to five consecutive frames, with an exposure time of 1 s per frame. No radiation-induced sample damage was observed during the X-ray exposure. Nanopure deionized water or filtered D_2_O was independently measured in the same sample holder for solvent subtraction. Data reduction and background subtraction were performed by using the Irena SAS macros for Igor Pro.^48^

Additional SAXS/WAXS measurements were performed at the Life Science X-ray Scattering (LiX/16-ID) beamline of the National Synchrotron Light Source II (NSLS-II), Brookhaven National Laboratory, and the 1W1A Diffuse X-ray Scattering Station, Beijing Synchrotron Radiation Facility (BSRF). The X-ray wavelength at LiX/16-ID and 1W1A was set to 0.827 and 1.54 Å, respectively, which is equivalent to X-ray energy of 15.0 and 8.05 keV, respectively. At the LiX beamline, three Pilatus detectors were used simultaneously, positioned at sample-to-detector distances of 3.58, 0.71, and 0.34 m. This configuration covered an effective *q*-range of ∼0.006–3.0 Å^–1^. At the 1W1A beamline, an EIGER X 1 M area detector (pixel size = 72 μm × 72 μm) was positioned at sample-to-detector distance of 0.113 m. This configuration covered an effective *q*-range of ∼0.15 –2.2 Å^–1^. Samples were loaded into a static liquid cell that has a path length of 1.5 mm capped with mica windows (∼20 μm thick, Richard Jahre GmbH, Wilhelmshaven, Germany). At the LiX beamline, the liquid cell is connected to a Peltier temperature controller for in situ high-temperature measurements. The final X-ray curves for each sample were obtained by averaging five consecutive frames, with an exposure time of 1 s (LiX) or 6 s (1W1A) per frame. Nanopure deionized water or filtered D_2_O was independently measured in the same sample holder for solvent subtraction. The data reduction was performed either by using the Python package py4xs available on GitHub (https://github.com/NSLS-II-LIX/py4xs) or by using the Nika 2D SAS macros for Igor Pro.^49^ No attempt was made to convert the WAXS data to an absolute scale.

## Results and Discussion

### Structural Evolution of A_100_M_101_D_10_ Triblock Terpolypeptoid Hydrogels

**Figure 1:**
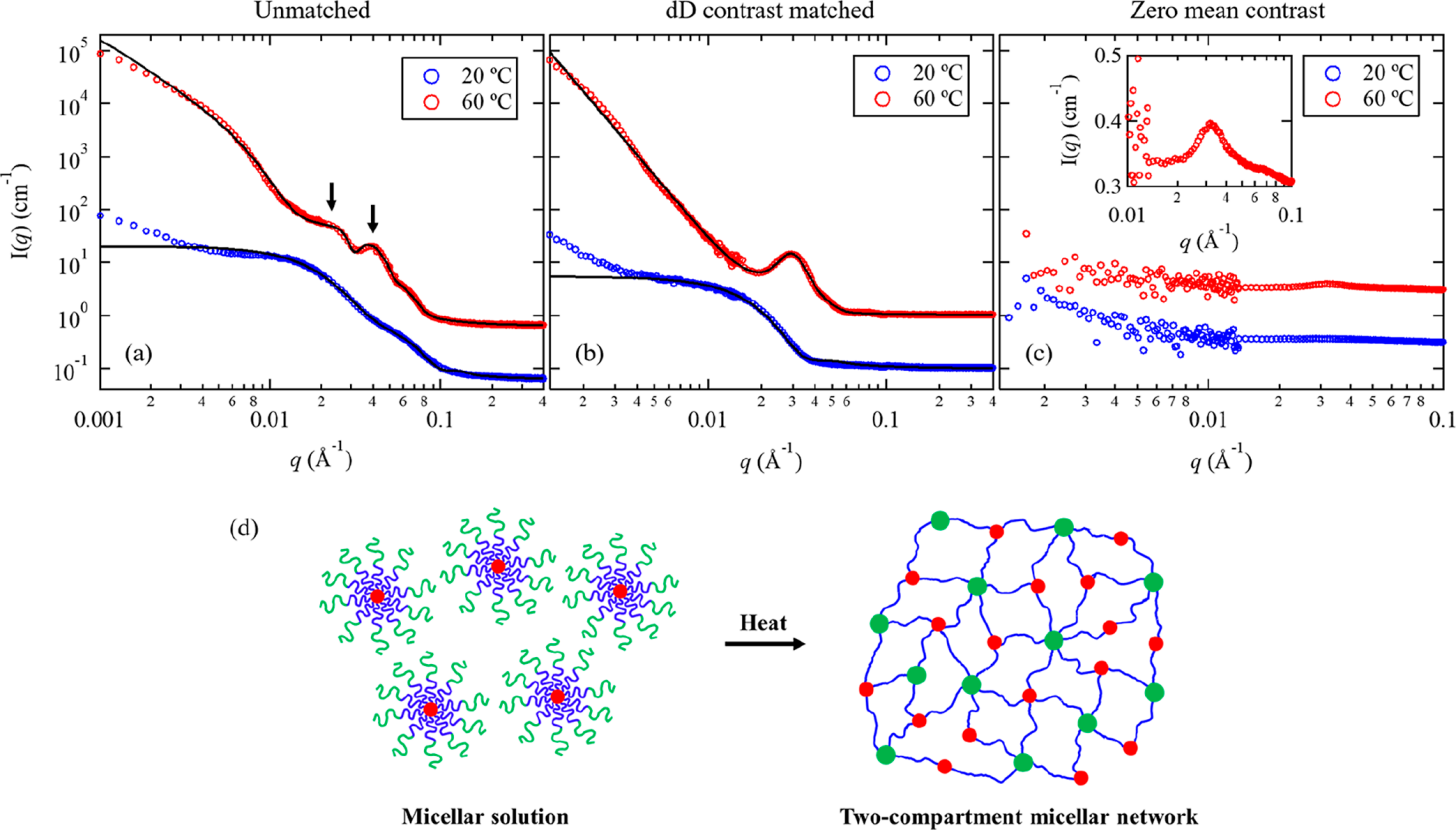
SANS profiles of (a) 1 wt % A_100_M_101_D_10_ in D_2_O, (b) 1 wt % A_95_M_99_dD_9_ in D_2_O/H_2_O with 93.8 vol % D_2_O (dD contrast matched condition), and (c) 1 wt % A_95_M_99_dD_9_ in D_2_O/H_2_O with 37.8 vol % D_2_O (zero mean contrast condition) measured at 20 and 60 °C. The solid black curves correspond to the best fits of the data using the models described in the text. The two scattering peaks in (a) are indicated by black arrows. All profiles were shifted vertically for clarity by multiplying a factor of 10. The corresponding SANS profile for the 1 wt % A_95_M_99_dD_9_ in D_2_O/H_2_O with 37.8 vol % D_2_O measured at 20 and 60 °C in linear intensity scale near *q* = 0.03 Å^–1^ is shown in the inset of (c). (d) Schematic illustration of the thermal-induced gelation of A_95_M_99_dD_9_ triblock terpolymers.

We first focus on the formation of AMD hydrogels with targeted composition of A_100_M_100_D_10_, which exhibit a sharp sol–gel transition from a free-flowing solution to a freestanding opaque gel at ∼27 °C at a solution concentration of 1 wt %.^31^Figure 1 shows the SANS intensity profiles of 1 wt % A_100_M_101_D_10_ in D_2_O measured at 20 °C (*T* < *T*_gel_) and 60 °C (*T* ≫ *T*_gel_) during the heating cycle. At 20 °C, the SANS profile exhibits typical scattering characteristic of a core–corona form factor, along with a Guinier plateau of around 0.01 Å^–1^. Cryo-TEM imaging of the 1 wt % A_100_M_101_D_10_ solution vitrified at 20 °C reveals the presence of spherical micelles with an average diameter of 6.5 ± 0.5 nm (Figure S16). Given that both the A end-block and M midblock are solvophilic at 20 °C,^31,32,50^ while the D block possesses hydrophobic *n*-decyl side chains,^31,40^ it is reasonable to conclude that A_100_M_101_D_10_ molecules self-assemble into spherical micelles at 20 °C, consisting of a hydrophobic D_10_ core and an outer shell-corona region composed of the A_100_M_101_ segments. The SANS profile for A_100_M_101_D_10_ in D_2_O at 20 °C was fitted using the scattering model for spherical polymer micelles developed by Pedersen and co-workers,^51−53^ in which the total coherent scattering is described in terms of a core–corona micelle form factor and a monodisperse hard-sphere structure factor. The detailed description of the theoretical scattering model was summarized in the Supporting Information. In brief, the scattering form factor for a spherical core–corona micelle are expressed in terms of the core radius (*R*_c_) and the radius of gyration of the Gaussian corona chains (*R*_g,chain_), where the corona chains are centered at a distance *d*_int_*R*_g,chain_ away from the surface of the core (*d*_int_ is close to unity to mimic nonpenetration of the corona chains into the core region). The monodisperse hard-sphere structure factor is expressed as a function of the hard-sphere radius (*R*_HS_) and the hard-sphere volume fraction of micelles (η_HS_).^54^ An *q*-independent term accounting for the incoherent scattering of D_2_O and hydrogen atoms in the polymer at high *q* was also incorporated in the fitting model. Note that a slight intensity upturn was observed at the very low *q* region (*q* < 0.004 Å^–1^), likely due to the clustering or secondary aggregation of A_100_M_101_D_10_ micelles on a larger scale. However, this feature was not considered during the data fitting.

In the fitting of SANS data, four independent parameters could be adjusted: *R*_c_, *R*_g,chain_, *R*_HS_, and η_HS_. The *d*_int_ value is fixed to 0.9 based on the previous study by Pedersen and Gerstenberg,^51^ assuming a relatively sharp core–corona interface where the coronal chains do not strongly penetrate into the core domain. This choice is justified by the distinct hydrophobic nature of the D block relative to the A and M blocks,^31,40,44,55^ which is likely to induce a strong segregation between the core and corona regions, as well as the good fits to our SANS data set. According to the bulk densities of poly(*N*-allyl glycine) and poly(*N*-methyl glycine) homopolymers,^39,56^ the neutron scattering length densities (SLDs) of the A (ρ_A_ = 1.84 × 10^–4^ nm^–2^) and M (ρ_M_ = 1.95 × 10^–4^ nm^–2^) blocks were found to be similar. Therefore, it is possible to use an average scattering length density to account for the outer A_100_M_101_ corona chain (SLD_chain_) during the model fitting. The scattering length density of the core-forming D block, (SLD_core_ = 0.258 × 10^–4^ nm^–2^) estimated based on the bulk density of poly(*N*-decyl glycine) homopolymer,^35,39,55^ was kept constant during the fitting procedure. The best fit to the data (Figure 1a) gives *R*_c_ = 4.5 ± 0.2 nm, *R*_g,chain_ = 5.0 ± 0.2 nm and *R*_HS_ = 6.1 ± 0.2 nm. The average aggregation number (*N*_agg_) of polymer chains in a single micelle of A_100_M_101_D_10_ is then estimated to be 92 by using *N*_agg_ = 4π*R*_c_^3^/3*V*_core_, where *V*_core_ is the molecular volume of a single D chain in the core. It should be noted that the *R*_HS_ value determined by the structure factor analysis is smaller than the micellar radius (*R*_mic_ = 15.5 ± 0.2 nm) estimated by a simple geometrical relationship *R*_mic_ = *R*_c_ + 2*R*_g,chain_. This indicates that the corona-forming A_100_M_101_ block can swell significantly in D_2_O, which allows the coronal regions of adjacent micelles to interpenetrate, causing the overall micellar radius to be larger than the hard-sphere radius.

Upon heating to temperatures above *T*_gel_, the A_100_M_101_D_10_ micellar solution undergoes a sol–gel transition, transforming into a freestanding hydrogel. This transition results in a drastic change in the scattering profile. The SANS profiles of 1 wt % A_100_M_101_D_10_ in D_2_O measured at 60 °C (*T* ≫ *T*_gel_) shows two distinct scattering peaks near *q* = 0.022 and 0.040 Å^–1^ along with a significant intensity increase at the low-*q* region (Figure 1a). This feature indicates the formation of a physically cross-linked hydrogel network comprised two different compartmental domains: a dehydrated A domain and a hydrophobic D domain. As illustrated in Figure 1d, the A and D domains are not randomly packed in the hydrogel, instead, they are alternatingly arranged, with each type of sphere surrounded by the other type. The hydrophilic M midblock serves as a bridging component in the network, connecting the A and D compartments. This configuration results in two characteristic length scales in the spatial correlation, yielding two distinct scattering peaks.

A scattering model previously proposed by Lodge and coworkers^22^ that describes the intermicellar correlation within the two-compartment network of ABC-type hydrogels was considered for the SANS data analysis. In brief, the model includes form factors for binary spheres and structure factors that describe the sticky hard-sphere interactions among spheres, which is given by the equation^22^
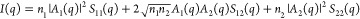
1where *n*_1_ and *n*_2_ are the number densities of the two types of spheres. The form factor amplitudes for binary hard spheres, *A*_1_(*q*) and *A*_2_(*q*), are determined by multiple parameters including the total volume fraction of the polymer, the hard-sphere volume fractions, micellar radii, interfacial widths of the two types of spheres and their SLD contrasts with the surrounding medium. In the equation, *S*_11_(*q*), *S*_12_(*q*), and *S*_22_(*q*) are the sticky hard-sphere structure factors that describe the interactions between type-1 and type-2 spherical domains, considering both repulsive and attractive interactions. The attractive strength and range of attraction between particles are described by a “stickiness” parameter τ, which is expressed in terms of the hard-sphere diameter, as well as the depth and width of a square-well potential.^22,57−60^ The detailed description of the scattering model was summarized in the Supporting Information.

In addition to the binary sticky hard-sphere model, the SANS profile for the A_100_M_101_D_10_ hydrogel also displays a strong low-*q* upturn arises from heterogeneously distributed polymer-rich gel phase in the network at large length scales. Hence, an exponential decay function was implemented in the scattering model to empirically fit the large-scale spatial heterogeneities of the polymeric networks.^22^ In addition, a high-*q* scattering contribution from the bridging M chains and an *q*-independent incoherent scattering term (*I*_inc_) were also included. The total scattering intensity for the AMD hydrogel is therefore given by
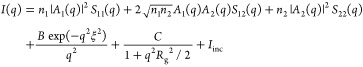
2where the fourth term is an exponential decay function multiplied by 1/*q*^2^ and the fifth term is the Debye-type function that describes the scattering contribution from bridging chains.^22,61^ Here, *B* and *C* are coefficients that are proportional to the number density of the scattering objects and their squared scattering length density contrast against water, ξ is the correlation length of the spatial heterogeneity, which can be interpreted as the average mesh size of the hydrogel network, and *R*_g_ is the radius of gyration of the bridging chains. The individual scattering contributions from each term in eq 2 are displayed in Figure S17.

To further validate the proposed scattering model, we conducted contrast variation SANS experiments on AMD hydrogel samples comprised a partially deuterated D block, that is, poly(*N*-allyl glycine)-*b*-poly(*N*-methyl glycine)-*b*-deuterated poly(*N*-decyl glycine) (AMdD). The partially deuterated AMdD polymer was similarly synthesized by sequential ROP method using the deuterated De-NCA monomer and was found to have comparable composition as the fully hydrogenated counterparts (Table 1). Based on the contrast factor calculations (Figure S18), two different contrast matching conditions, namely “dD contrast matched condition” and “zero mean contrast condition,” can be achieved by adjusting the D_2_O/H_2_O ratio. We first focus on the dD contrast matched condition, where the micellar core that composed of dD block becomes practically “invisible” under neutron scattering, and the scattering is dominated by the thermal-responsive A block. Figure 1b shows the SANS profiles for the 1 wt % A_95_M_99_dD_9_ in D_2_O/H_2_O with 93.8 vol % volume fraction of D_2_O measured at 20 and 60 °C. At *T* < *T*_gel_, the typical scattering oscillation near *q* = 0.04 Å^–1^ for a core–corona type structure was barely discernible as the dD block is contrast-matched out. At *T* ≳ *T*_gel_, there is only a single scattering peak at *q* ≈ 0.03 Å^–1^ that mainly arises from scattering contribution of dehydrated A spheres. Since A_100_M_101_D_10_ and A_95_M_99_dD_9_ have similar composition, the SANS profiles at the unmatched and the dD contrast-matched condition were fitted simultaneously by setting constrained parameters. This simultaneous fitting process allows us to obtain the structural parameters of the hydrogel network, particularly the dehydrated A domains, with less uncertainty as compared to those obtained by conducting the fitting independently.

For the SANS data of hydrogels measured at 60 °C, eight independent parameters could be adjusted to obtain the best fits: the core radius of A domain (*R*_c,A_), the interfacial widths of A and D domains (ω_A_, ω_D_), the hard-sphere volume fraction of A and D domains (*f*_A_, *f*_D_), the volume fraction of water in A (η_water,A_), the volume fraction of water within the gel phase (η_water,gel_), the interparticle “stickiness” between A and D domains (τ), the interparticle hard-sphere diameter (*d*_hs_) between A and D, the prefactors B and C associated with the low-*q* and high-*q* terms, and the correlation length of the spatial heterogeneity of the network (ξ). Since the hydrophobic D block is nonthermally responsive, the core radius of D (*R*_c,D_ = 4.5 ± 0.2 nm) determined by SANS fitting of the AMD micelles at 20 °C was kept constant for all temperatures. Note that the *R*_g_ of the bridging chains (M) was fixed at 3.5 nm, which falls between the *R*_g_ of the M_100_ homopolymer in water (2.6 nm, as determined through a separate SAXS measurement) and half of the edge-to-edge distance between A and D micellar domains (∼4.0 nm) as determined by SANS analysis. In fact, for the dD contrast matched condition, we found that the low-*q* upturn observed in Figure 1b can be better fitted by a Debye–Bueche term^62,63^ rather than an exponential decay term described in eq 2 (Figure S19). Hence, the total scattering intensity for the AMD hydrogel under the dD contrast matched condition is given by
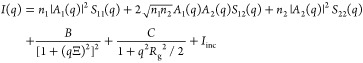
3where the fourth term is the Debye–Bueche inhomogeneity function expressed by a squared Lorentzian function, which gives a Porod-like *I*(*q*) ∼ *q*^–4^ behavior at large *q*. Here, Ξ represents the correlation length of the spatial heterogeneity of the hydrogel network under the dD contrast matched condition. The difference in the low-*q* scattering terms in eqs 2 and 3 reflects the distinct types of structural heterogeneities between polymer-rich and polymer-poor region observed under the under “unmatched condition” and “dD contrast matched condition.” Note that the difference in the description of the low-*q* term has negligible effect on the other fitting parameters obtained from the binary sticky hard-sphere contribution listed in Table 2.

**Table 2:** Fitting Parameters for AMD Hydrogels at 60 °C

fitting parameters	A_100_M_101_D_10_ in D_2_O	A_95_M_99_dD_9_ in D_2_O/H_2_O (dD contrast matched)^a^	A_100_M_110_dD_18_ in D_2_O/H_2_O (dD contrast matched)^a^
*R*_c,A_ (nm)	7.2	7.2	7.7
ω_A_ (nm)	1.2	1.2	0.5
*R*_c,D_ (nm)^b^	4.5	N/A	N/A
ω_D_ (nm)	2.3	N/A	N/A
η_water,gel_ (%)^c^	55	55	49
η_water,A_ (%)^d^	14	14	13
*n*_A_/*n*_D_^e^	0.54	N/A	N/A
τ^f^	5	N/A	N/A
ξ (nm) or Ξ (nm)^g^	∼14	∼439	∼44

a The volume fraction of D_2_O in the D_2_O/H_2_O mixture is 93.8%.

b The *R*_c,D_ value is fixed at 4.5 nm, which is determined from SANS analysis of the A_100_M_101_D_10_ solution at 20 °C.

c The fraction of D_2_O or D_2_O/H_2_O content within the gel phase.

d The fraction of D_2_O or D_2_O/H_2_O content within the dehydrated A domain.

e The number ratio between A and D micellar cores in the binary system determined by eqs S18 and S19 in the Supporting Information.

f Interaction potential between A and D domains in the sticky hard-sphere structure factor *S*_12_(*q*). Note that the error bar for *R*_c,A_, *R*_c,D_, ω_A_ and ω_D_ obtained from the model fitting is ±0.1 nm; likewise, the error bar for η_water,gel_ and η_water,A_ is ±1.5%.

g ξ (nm) and Ξ (nm) represent the correlation lengths of the spatial heterogeneity described by eqs 2 and 3, respectively.

Table 2 summarizes the structural parameters of A_100_M_101_D_10_ in D_2_O and A_95_M_99_dD_9_ in D_2_O/H_2_O under dD contrast matched condition at *T* = 60 °C obtained from the best fits to the data (Figure 1). The dehydrated A micellar core has a radius (*R*_c,A_) of 7.2 nm and contains 14% of water content, indicating A is not fully dehydrated. The number fraction, i.e., total number of A cores relative to D cores (*n*_A_/*n*_D_), was estimated to be ∼0.54. This suggests that the D block forms nearly twice more junction points than the A block in the two-compartment network. In the structure factor *S*_12_(*q*), the hard-sphere diameter, which describes the distance below that A and D domains cannot come any closer due to repulsive interactions, was estimated to be 39.2 nm. The ξ value by model fitting the unmatched condition was estimated to be ∼14 nm, most likely corresponding to the mesh size or the minimum intermicellar distance within the network. On the other hand, the correlation length (Ξ) obtained from eq 3 under dD contrast matched condition was ∼439 nm, indicating a characteristic length of inhomogeneities much larger than the smallest mesh size.

It is worth noting that at 60 °C, the primary peak position of A_95_M_99_dD_9_ in D_2_O/H_2_O (*q* ≈ 0.03 Å^–1^) differs slightly from that of A_100_M_101_D_10_ in D_2_O (*q* ≈ 0.022 Å^–1^). Despite their similar chemical composition, this difference primarily arises from the scattering length density contrast (Δρ) between the polymer blocks and water. Here, Δρ^2^ is the prefactor in SANS intensity without considering specific size, shape, or structural correlations (*I* ∼ Δρ^2^). In the case of AMD triblock terpolymers in D_2_O, the estimated Δρ^2^ value for a fully collapsed D core (3.76 × 10^21^ cm^–4^) is more than three times greater than that of a partial dehydrated (i.e., η_water,A_ = 25%) A core (1.16 × 10^21^ cm^–4^) (Figure S18). Consequently, we postulate that the primary scattering peak located at *q* ≈ 0.022 Å^–1^ in Figure 1a predominantly arise from the spatial correlation among D cores, whereas under dD contrast matched condition, the single scattering peak primarily originates from the scattering contribution of the dehydrated A cores. These SANS results provide direct experimental support for the formation of a two-compartment hydrogel network comprised of thermoresponsive AMD triblock terpolypeptoids, as proposed in the previous study.^31^

Figure 1c shows the SANS profiles of 1 wt % A_95_M_99_dD_9_ in D_2_O/H_2_O with 37.8 vol % D_2_O measured at 20 and 60 °C under the zero mean contrast condition, where the SLD of the D_2_O/H_2_O mixture is made equal to the averaged SLD of the AMdD polymer. This condition results in minimal scattering from the entire triblock terpolymer, while the contribution from individual blocks can still be identified when there is sufficient contrast with their surroundings. Hence, the low-*q* intensity upturn that arises from the spatial inhomogeneity between polymer-rich and polymer-poor region become absent at *T* > *T*_gel_ (Figure 1c), while the scattering peak that arises from the spatial correlation of micellar cores is still observable (inset of Figure 1c). The presence of a flat low-*q* region in the SANS profile is also consistent with the lack of elongated domains within the two-compartment network.

**Figure 2:**
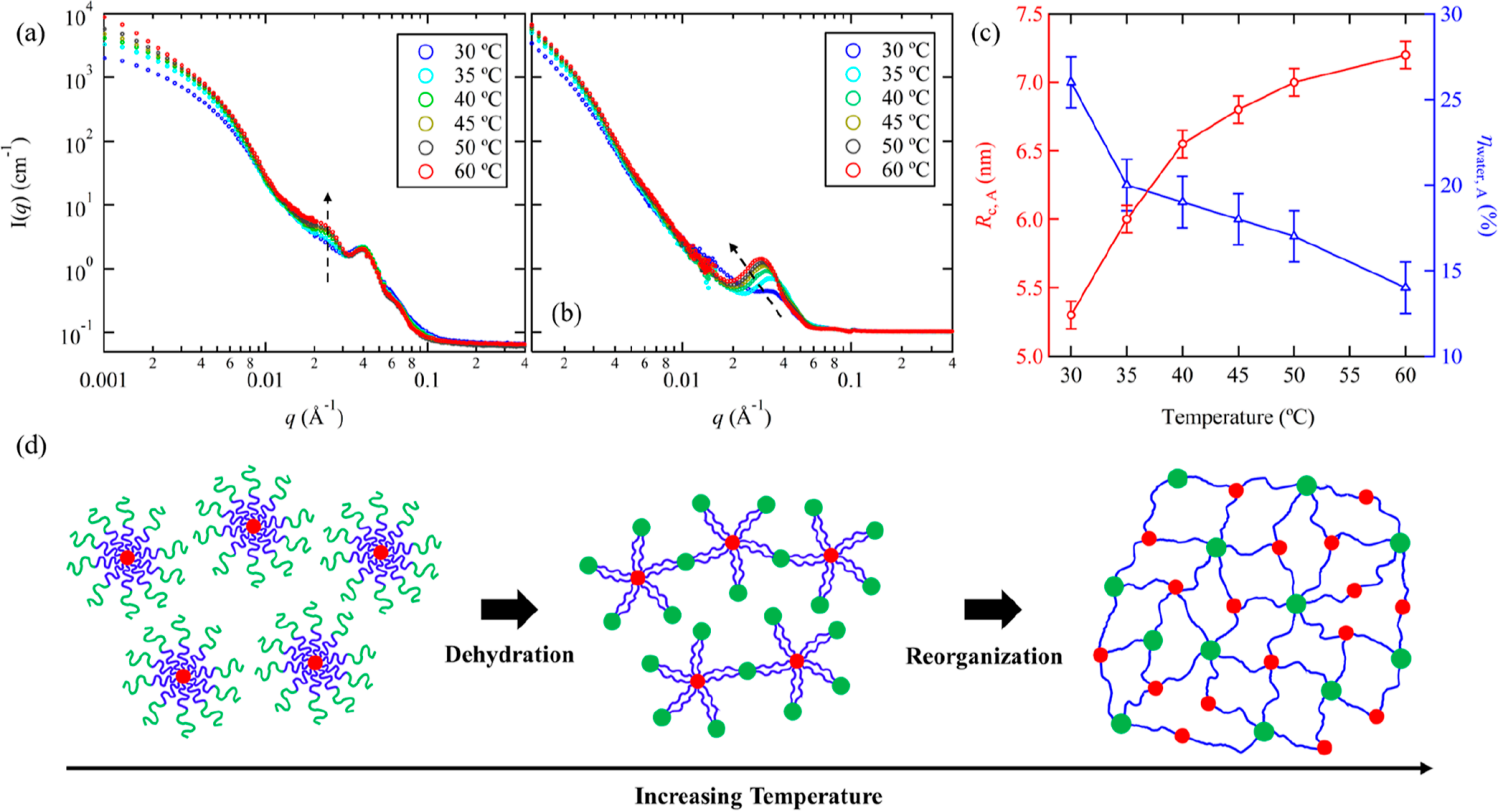
SANS profiles of (a) 1 wt % A_100_M_101_D_10_ in D_2_O and (b) 1 wt % A_95_M_99_dD_9_ in D_2_O/H_2_O with 93.8 vol % D_2_O (dD contrast matched condition) measured during heating process at *T* ≳ *T*_gel_. (c) Changes of core radius (*R*_c,A_) and fraction of water content (η_water,A_) of A domain as a function of temperature. (d) Schematic illustration of the proposed structural change of AMD spherical micelles during temperature-induced sol–gel transition.

Figure 2a shows the temperature dependence of the SANS profiles for A_100_M_101_D_10_ in D_2_O at *T* ≳ *T*_gel_. At *T* = 30 °C, which is close to *T*_gel_, only one peak near *q* = 0.040 Å^–1^ is observed, and no scattering peak near *q* = 0.022 Å^–1^ is discernible. Based on the contrast factor calculation and SANS analysis for the dD contrast matched condition, this secondary peak is attributed to the correlation distance between A and D domains connected by the bridging M block. As the temperature further increases, the primary scattering peak near *q* = 0.022 Å^–1^ emerges, followed by a gradual increase in peak intensity, while the secondary peak remains unchanged throughout the heating process. In situ small-angle X-ray scattering (SAXS) measurements further confirm that the change in the primary peak near *q* = 0.022 Å^–1^ is dependent on temperature rather than time under isothermal conditions (Figure S20). We also confirm that the scattering peak positions remain unchanged with increasing polymer concentration to 5 wt % (Figure S20). The increasing intensity of the primary peak in Figure 2a indicates a gradual enhancement of the intermicellar ordering of D domains with increasing temperature. On the other hand, for the temperature-dependent SANS curves of A_95_M_99_dD_9_ in D_2_O/H_2_O (dD contrast-matched condition), where the dehydrated A cores dominate the scattering intensity in the mid-*q* region, the single scattering peak gradually intensifies and shifts toward lower *q* with increasing temperature (Figure 2b). Based on the best fits of the SANS data under dD contrast matched condition using eq 3 (Figure S21), it was found that *R*_c,A_ gradually increases from 5.3 ± 0.1 nm (at 30 °C) to 7.2 ± 0.1 nm (at 60 °C), while η_water,A_ decreases from 26.0 ± 1.5% (at 30 °C) to 14.0 ± 1.5% (at 60 °C) due to temperature-induced dehydration (Figure 2c). By excluding the volume occupied by water and using the theoretical molecular volume of the A block, the aggregation numbers of spherical A domain (*N*_agg,A_) were estimated to be 28 ± 1 (at 30 °C) and 82 ± 1 (at 60 °C), respectively. These observations indicate that the formation of the two-compartment hydrogel network of AMD is not a single-step process but involves a continuous structural reorganization coupled with the temperature-dependent dehydration of the A block. During the heating process, the A spheres continue to dehydrate and, simultaneously, enlarge as more A blocks aggregate or assemble into the same domain. Due to the molecular connectivity of A and D blocks, the temperature-induced aggregation of A blocks also facilitates the reorganization of the D domains, leading to interconnected micelles and the formation of well-defined two-compartment network in the aqueous solution (Figure 2d).

### Effect of Chain Length of the Core-Forming D Block on Structural Evolution

**Figure 3:**
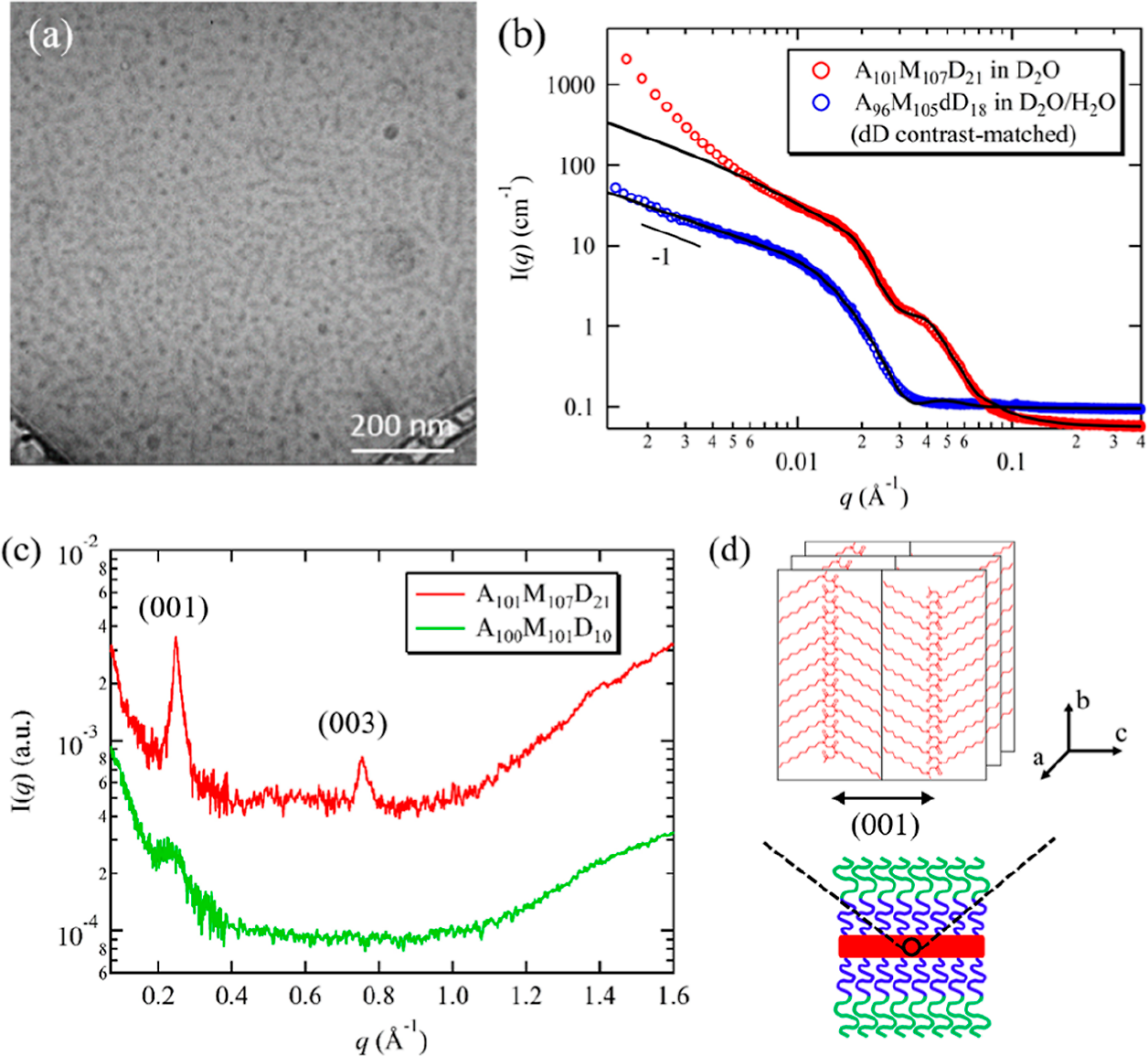
(a) Representative cryo-TEM images of 1 wt % A_101_M_107_D_21_ solution vitrified at 40 °C (*T* > *T*_gel_). (b) SANS profiles of 1 wt % A_101_M_107_D_21_ in D_2_O and A_96_M_105_dD_18_ in D_2_O/H_2_O with 93.8 vol % D_2_O (dD contrast-matched condition) measured at 20 °C (*T* < *T*_gel_). The solid lines correspond to the best fit of the SANS the data using the polymeric micelle model with a rod-shaped core. (c) WAXS profile of 1 wt % A_100_M_101_D_10_ and A_101_M_107_D_21_ in D_2_O measured at 20 °C. (d) Schematic illustration of the molecular packing of core-forming D blocks. Note that this illustration does not specify the detailed crystalline orientation within the micellar core.

According to previous studies,^33,37,38,40−42^ polypeptoids with relatively long *n*-alkyl side chains are capable of crystallization and exhibit two ordered phases: a highly ordered orthorhombic crystalline phase and a “sanidic” liquid crystalline (LC) mesophase when cooled from the isotropic melt. In the crystalline phase, polypeptoid molecules adopt a board-like motif where the backbone is fully extended in an all *cis*-amide conformation and is approximately coplanar with the *n*-alkyl side chains.^37,38,41,42^ As discussed above, in the case of AMD with a small volume fraction of D, such as A_100_M_101_D_10_, the triblock terpolymers form spherical micelles at *T* < *T*_gel_. WAXS analysis of the A_100_M_101_D_10_ micellar solution and hydrogel revealed the absence of any diffraction peak (Figure 3c), consistent with the lack of any molecular ordering in the D domains. Interestingly, as the volume fraction of D increases, there is a notable change of D domain morphology due to the molecular packing of D block to form ordered LC phases. Cryo-TEM analysis (Figure 3a) for the 1 wt % A_101_M_111_D_21_ hydrogel sample revealed the coexistence of both spherical domains and elongated rod-like domains. The rod-like particles exhibit an average length of ∼50 nm and a diameter of ∼10 nm. Note that similar rod-like micelles were also observed in the corresponding micellar solution vitrified at *T* < *T*_gel_.^31^

Figure 3b shows the SANS profiles for 1 wt % A_101_M_107_D_21_ in D_2_O (unmatched condition) and A_96_M_105_dD_18_ in D_2_O/H_2_O with 93.8% D_2_O (dD contrast-matched condition) measured at 20 °C (*T* < *T*_gel_). For the unmatched condition, the SANS intensity profile for A_101_M_107_D_21_ micellar solution exhibits scattering feature of core–corona type micelles at mid-*q* region and a significant intensity upturn at low-*q*. When the scattering contribution of the core-forming D block is contrast matched, the SANS intensity profile gives a power law dependence on *q* with a −1 exponent at the low-*q* region. Hence, it is reasonable to fit the data by using the scattering form factor of a polymeric micelle comprised of a cylindrical rod-shaped core surrounded by outer corona chains (see the Supporting Information for details).^51,52^ For the unmatched condition, a hard-sphere structure factor was included to approximate the intermicellar interactions. Note that this is only a rough approximation and may not fully represent the actual interactions of elongated micelles.^64^ For A_101_M_107_D_21_ in D_2_O, we speculate that the deviation from the *I*(*q*) ∼ *q*^–1^ relationship in the low-*q* region may be attributed to the secondary micellar aggregation or the presence of micelles with irregularly elongated core due to the allowed two-dimensional molecular packing of the D block. Based on the best fits of the SANS data under unmatched condition (i.e., in D_2_O) using cylindrical core–corona micelle model, the radius of the cylindrical core (*R*_c_) and the *R*_g,chain_ values are estimated to be 5.0 ± 0.1 and 7.7 ± 0.1 nm, respectively. The radius of cylindrical micelle (*R*_mic_) is estimated to be 20.4 ± 0.2 nm via the *R*_mic_ = *R*_c_ + 2*R*_g,chain_ relationship. Note that the *R*_c_ value is comparable to the fully extended backbone chain length of D_21_ (6.3 nm) in an all *cis*-amide conformation.

The molecular packing of D block within the rod-shaped core is revealed by solution WAXS measurements. As shown in Figure 3c, the A_101_M_107_D_21_ aqueous solution exhibit a sharp diffraction peak at *q* = 0.248 Å^–1^, corresponding to a *d*-spacing of 2.5 nm via the *d* = 2π/*q* relationship. This *d*-spacing corresponds to the distance between adjacent backbones of D segments that are separated by the long *n*-decyl side chains along the crystallographic *c*-axis,^33,37−39^ as illustrated in the inset of Figure 3d. A diffraction peak near *q* = 0.744 Å^–1^ is also observed, attributed to the (003) reflection. Note that the (002) reflection, which is expected to be located at 0.496 Å^–1^, is barely discernible, likely due to the high symmetry of the lamellar structure in the crystalline lattice. At higher *q*, we observed a broad peak near *q* = 1.38 Å^–1^, corresponding to the closest packing distance between D block backbones along the crystallographic *a*-axis of the board-like crystalline structure (Figure 3d) with a *d*-spacing of 0.455 nm.^37−40^ However, higher order (10*l*) reflections, which are typically observed for highly ordered poly(*N*-decyl glycine) (D) crystals,^33,37−40^ are absent. The above-mentioned results suggest that the core-forming D block stack into the “sanidic” LC mesophase inside the micellar core in aqueous solution,^37,41^ where the molecular ordering of the D blocks is short ranged. Based on temperature-dependent WAXS measurements, it was found that the (001) peak for the D_21_ block remains almost unchanged from 20 to 60 °C (Figure S22). Hence, the morphology and molecular packing of the rod-shaped D core is nonthermal responsive and remain unchanged upon thermally induced gelation. Due to the LC packing driven by strong intermolecular forces (e.g., dipole–dipole interaction along the D backbone) and hydrophobic interactions, it is expected that the molecular exchange is largely restricted due to high free energy penalty. This stabilizes the morphology of D cores, making them a thermally stable compartment in the hydrogel network.

**Figure 4:**
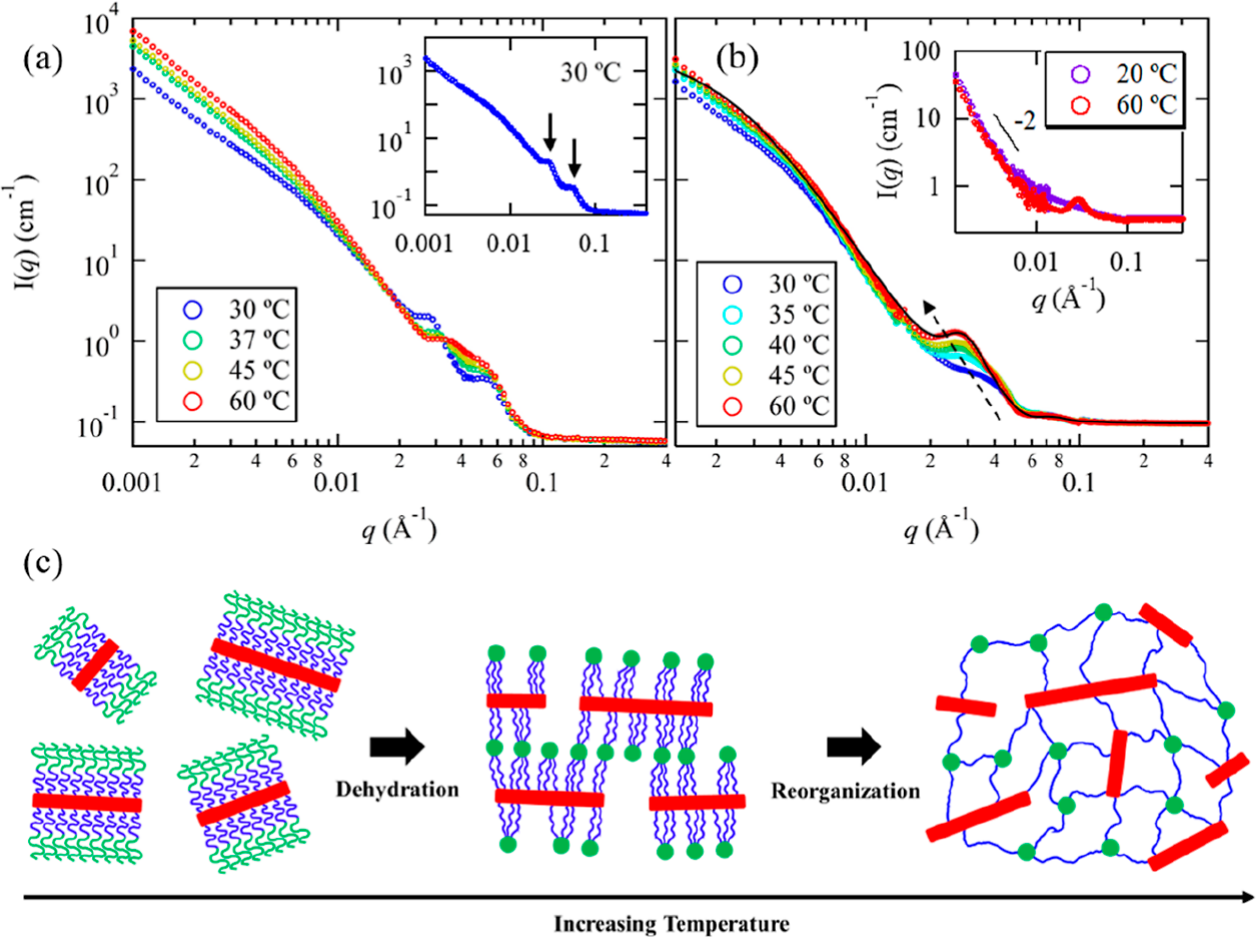
SANS profiles of 1 wt % (a) A_101_M_107_D_21_ in D_2_O and (b) A_96_M_105_dD_18_ in D_2_O/H_2_O with 93.8 vol % D_2_O (dD contrast-matched condition) measured during heating process at *T* ≳ *T*_gel_. The inset of (a) shows the SANS profile of A_101_M_107_D_21_ in D_2_O measured at 30 °C to highlight the two scattering peaks, as indicated by the black arrows. The inset of (b) shows the SANS profiles of A_96_M_105_dD_18_ in D_2_O/H_2_O with 40.0 vol % D_2_O (zero mean contrast condition) measured at 20 and 60 °C. (c) Schematic illustration of the proposed structural change of AMD micelles with an elongated LC-like D core during temperature induced sol–gel transition.

Figure 4 shows the SANS profiles for the 1 wt % A_101_M_107_D_21_ hydrogel in D_2_O and the A_96_M_105_dD_18_ hydrogel in D_2_O/H_2_O with 93.8% D_2_O (i.e., dD contrast-matched condition) measured at different temperatures at *T* ≳ *T*_gel_. For the A_101_M_107_D_21_ hydrogel, the SANS profile measured at 30 °C exhibits two distinct scattering peaks located near *q* = 0.027 Å^–1^ and *q* = 0.05 Å^–1^, respectively, suggesting the formation of two-compartment network. With increasing temperature, the two peaks gradually change their positions and merge into one broad peak (or hump) located around *q* = 0.04 Å^–1^. This trend is notably different from that of the A_100_M_101_D_10_ hydrogel comprised of lower volume fraction of D (Figure 2a). Note that due to the coexistence of spherical A cores and rod-shaped D cores in A_101_M_107_D_21_ hydrogel, the corresponding scattering model for a two-compartment network would become much more complex. As a result, we did not attempt to conduct a global model fitting of the SANS data for the A_101_M_107_D_21_ hydrogel in D_2_O (Figure 4a). Under the dD contrast-matched condition, the temperature-dependent structural evolution of A block can be analyzed by model fitting the SANS profiles using eq 3. As shown in Figure 4b, the SANS profiles for A_100_M_110_dD_18_ show a single scattering peak at *T* ≳ *T*_gel_, which is attributed to the intermicellar distance between the dehydrated A cores in the hydrogel networks. Interestingly, from 30 to 60 °C, the scattering peak gradually intensifies and shifts toward lower *q* with increasing temperature. This trend is comparable to that observed for A_95_M_99_dD_9_ under dD contrast matched condition (Figure 2b). Based on the best fit of the SANS data measured at 60 °C, the *R*_c,A_, η_water,gel_ and η_water,A_ values for A_100_M_110_dD_18_ were estimated to be 7.7 nm, 49% and 13%, respectively, which are similar to those observed for A_95_M_99_dD_9_ (Table 2). The changes of *R*_c,A_ and η_water,A_ as a function of temperature obtained from the best fits of the data were summarized in Figure S23. These results indicate that crystallization of the D block into rod-shaped cores does not affect the core size, intermicellar distance, or hydration state of A domains. Within both noncrystalline A_95_M_99_dD_9_ and crystalline A_100_M_110_dD_18_ two-compartment hydrogel networks, the A blocks undergo similar temperature-dependent dehydration and rearrangement processes upon gelation, albeit the large morphological difference in the initial AMD micelles in solution (i.e., spherical-shape vs. rod-shape).

The effect of crystallization of core-forming D block on the two-compartment hydrogel network is revealed by comparing the temperature-dependent SANS profiles for A_100_M_101_D_10_ (Figure 2a) and A_101_M_107_D_21_ in D_2_O (Figure 4a) under unmatched condition. As aforementioned, the scattering peaks observed in SANS under unmatched condition predominantly arise from the spatial correlation among D cores due to the relatively large neutron scattering contrast (Δρ). For the A_100_M_101_D_10_ hydrogel, the temperature-dependent SANS profiles suggest that increasing of temperature at *T* ≳ *T*_gel_ leads to more ordered intermicellar structures among spherical D cores in the two-compartment hydrogel network, as evidenced by the emergence of the primary scattering peak at *q* = 0.022 Å^–1^ (Figure 2). In contrast, the SANS profiles for the A_101_M_107_D_21_ hydrogel measured at 30 °C, which is near *T*_gel_, exhibits two well-defined scattering peaks located near *q* = 0.027 Å^–1^ and *q* = 0.05 Å^–1^. Upon temperature elevation, the two peaks gradually smeared out and merged into a broad hump, indicating a gradual loss of ordering in the spatial arrangement among D cores (Figure 4). The effect of elongated crystalline micelles on temperature-induced structural evolution of AMD is depicted in Figure 4c and explained as follows. At *T* < *T*_gel_, the elongated rod-shaped AMD micelles with a crystalline D core possess a higher aggregation number (*N*_agg_) of polymer chains per micelle, as compared to the spherical ones. Thus, the rod-shaped micelles have a larger outer corona region per a single micelle that can undergo temperature-induced dehydration. Once the corona-forming A block begins to dehydrate and collapse, it generates new hydrophobic A domains on the exterior region of the rod-shaped micelles. The dehydrated A domains lead to aggregation of rod-shaped micelles due to hydrophobic interactions. Consequently, when the initial dehydration of the A block occurs, the rod-shaped micelles coated with the now-collapsed A-blocks aggregate, thereby resulting in an ordered packing of the D cores (Figure 4c). However, as the A blocks continue to dehydrate and rearrange themselves upon further increase in temperature, the rod-shaped D cores have to change their position to accommodate the two-compartment network. As a result, the two distinct scattering peaks shown in Figure 4a, which appear immediately upon gelation of A_101_M_107_D_21_ at 30 °C, gradually diminish in intensity with further heating to 60 °C.

The above-mentioned results indicate that initial formation of elongated micelles can significantly affect the thermoresponsive structural reorganization of the hydrogel network. Since more D segments are aggregated in a rod-shaped domain relative to a spherical one, it is expected that the A_101_M_111_D_21_ hydrogel has a smaller number of physical cross-links formed by the D cores relative to the A_100_M_101_D_10_ hydrogel at the same polymer concentration. While a detailed analysis of SANS data from the A_101_M_111_D_21_ hydrogel is difficult due to elongated D core, we can estimate the correlation length ξ corresponding to the average mesh size of the hydrogel network using the shape-independent low-*q* scattering function. By fitting the low-*q* upturn under unmatched condition, the ξ value of the A_101_M_111_D_21_ hydrogel is estimated to be ∼10 nm at 60 °C, which is ∼30% smaller than that of the A_100_M_101_D_10_ hydrogel. Under dD contrast-matched condition, the Ξ value for A_96_M_105_dD_18_ (∼44 nm) is also much less than that for A_100_M_101_D_10_ (∼439 nm) (Table 2), suggesting that the network has a much smaller correlation length. Therefore, at the same polymer concentration, introducing an elongated rod-shaped D domain would result in fewer cross-links and a smaller local mesh size, thereby leading to more fragmented network structure in the hydrogel. In other words, the AMD with a longer, crystallizable D block would require a higher polymer concentration to form a continuous network structure that spans the entire volume of the hydrogel.

### Effects of Chain Length of Thermoresponsive A Block on Structural Evolution

It is known that for block copolymer micelles, the thermodynamic equilibrium micellar morphology is governed by minimization of the overall free energy due to interfacial tension between the solvophobic blocks and the solvent as well as the entropy penalty associated with stretching of polymer chains anchored at the core–corona interface, and is therefore dependent on the composition of the block copolymers.^65^ When the hydrophilic corona-forming block is much longer than the core-forming D block, one would expect that the LC packing of D blocks can be suppressed due to large excluded volume interactions of hydrophilic blocks. On the other hand, if the hydrophilic corona-forming block is relatively short, the AMD polymers can assemble into elongated micelles driven by the mesogenic interactions between the D blocks and the hydrophobic effect. Accordingly, here we show how the chain length of thermoresponsive A block affects the initial micellar morphology and subsequent hydrogel network formation.

**Figure 5:**
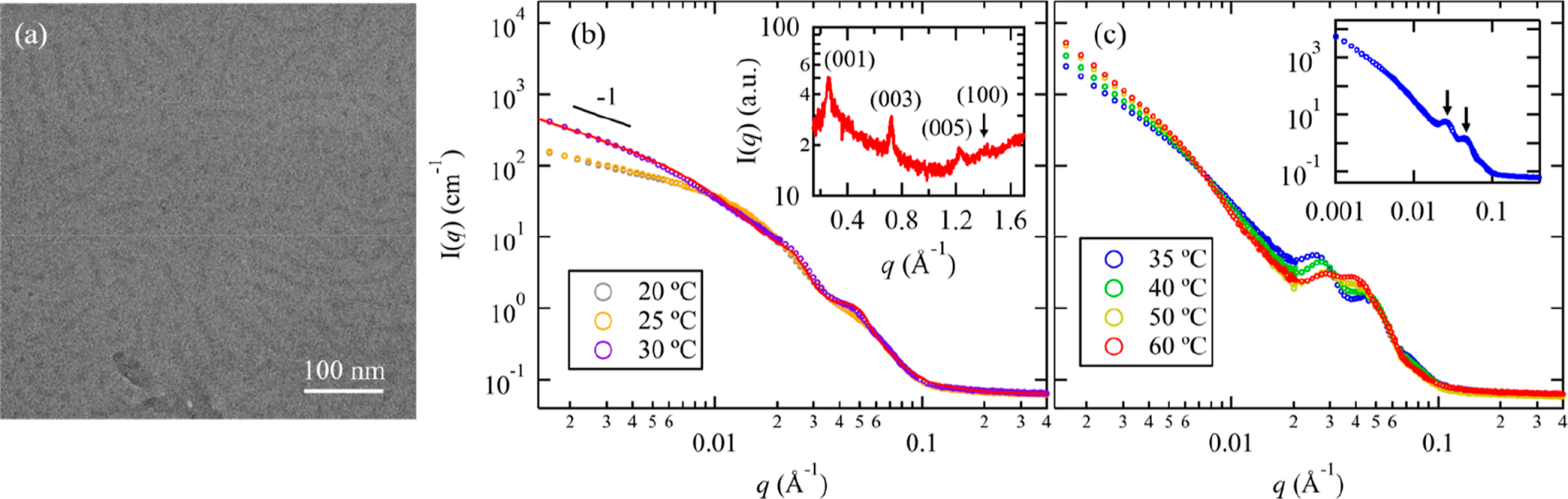
(a) Representative cryo-TEM images of 1 wt % A_43_M_92_D_9_ solution vitrified at 20 °C. (b,c) SANS profiles of 1 wt % A_43_M_92_D_9_ in D_2_O measured during heating process at (b) *T* < *T*_gel_ and (c) *T* ≳ *T*_gel_. The red solid line corresponds to the best fit of the SANS data measured at 30 °C based on the cylindrical polymer micelle model described in the text. The corresponding WAXS profile of 1 wt % A_43_M_92_D_9_ in D_2_O is shown in the inset of (b). The inset of (c) shows the SANS profile of A_43_M_92_D_9_ in D_2_O measured at 35 °C for clarity. The two scattering peaks due to spatial correlation among micelles are indicated by black arrows.

Figure 5a shows the cryo-TEM image the 1 wt % A_43_M_92_D_9_ solution vitrified at 20 °C, which revealed the presence of elongated rod-like micelles spanning several tens of nm in length and ∼6 nm in diameter. The temperature-dependent SANS profiles for 1 wt % A_43_M_92_D_9_ in D_2_O are shown in Figure 5b,c. An abrupt change in the SANS profiles from 30 to 35 °C was observed, suggesting a sol–gel transition. Note that the 5 wt % A_43_M_92_D_9_ solution exhibits similar structural evolution upon a temperature increase (Figure S24). Since A_43_M_92_D_9_ has a much shorter thermoresponsive A block compared to A_100_M_101_D_10_, it has a higher *T*_gel_ due to the higher cloud point of short A block.^31,32^ At *T* < *T*_gel_, the low-*q* region of the SANS profiles exhibits a power law dependence on *q* with an exponent close to −1, suggesting the formation of 1D elongated micelles. WAXS analysis of 1 wt % A_43_M_92_D_9_ micellar solution in D_2_O (inset of Figure 5b) indicates the LC packing of D block, in sharp contrast to the noncrystalline A_100_M_101_D_10_ micellar solution (Figure 3c). Hence, it is reasonable to fit the SANS profile of the A_43_M_92_D_9_ solution at *T* < *T*_gel_ using the aforementioned cylindrical core–corona micelle model.^51,52^ The best fit of the SANS data at 30 °C (Figure 5b) afforded *R*_c_ and *R*_g,chain_ to be 4.6 ± 0.1 and 7.3 ± 0.1 nm, respectively. The *R*_c_ value is consistent with the average radius of the rods obtained by cryo-TEM analysis (∼6 nm), indicating that only the core region of the micelle was visible in the cryo-TEM images. The estimated cross-sectional radius of cylindrical micelle (*R*_mic_ = *R*_c_ + 2*R*_g,chain_) is 19.2 ± 0.2 nm.

The above results clearly indicate that shortening the A block while retaining a relatively short D block in the AMD block copolymer can facilitate the formation of elongated micelles with liquid crystalline packing of the D block in the micellar core due to reduced contribution of the excluded volume interactions relative to the mesogenic interactions and hydrophobic interactions to the overall free energy of the micelles. At *T* ≳ *T*_gel_, the A_43_M_92_D_9_ hydrogel displays a temperature-dependent change in the SANS profiles similar to that observed for A_101_M_107_D_21_. As shown in the inset of Figure 5c, the 35 °C curve shows two sharp scattering peaks at *q* = 0.026 Å^–1^ and *q* = 0.046 Å^–1^, respectively. The two peaks gradually shift in *q* as temperatures increases and progress into two broad humps at 60 °C. Hence, when the AMD polymers form elongated micelles with a mesogenic core in aqueous solution initially, they display similar structural evolution upon sol–gel transition.

**Figure 6:**
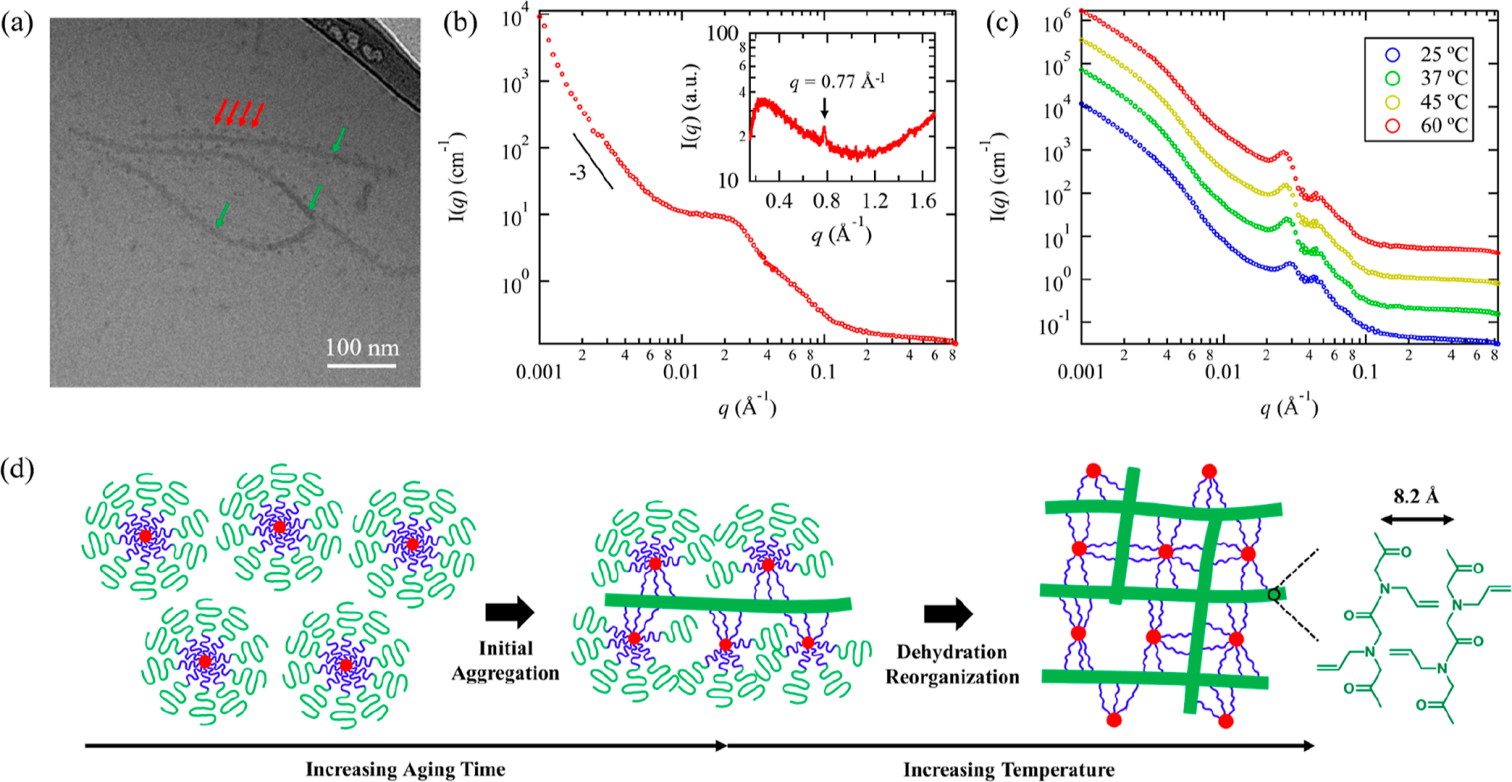
(a) Representative cryo-TEM image of 1 wt % A_153_M_127_D_10_ in aqueous solution vitrified from 20 °C. The fiber-like A domains and spherical D domains are indicated by green and red arrows, respectively. (b) SANS profile of the 5 wt % A_153_M_127_D_10_ in D_2_O measured at 20 °C (*T* < *T*_gel_). The corresponding WAXS profile of the sample (2.5 wt %) is shown in the inset of (b). The diffraction peak corresponds to the molecular packing of A block is indicated by the black arrow. (c) SANS profiles of 5 wt % A_153_M_127_D_10_ in D_2_O measured during heating process. Data were shifted vertically for clarity by multiplying a factor of 5. (d) Schematic illustration of the proposed structural change of AMD micelles with an elongated A domain during temperature induced sol–gel transition.

On the other hand, if the chain length of thermoresponsive A block is sufficiently long, it led to a very different scenario. It was previously reported that the cloud point temperature of poly(*N*-allyl glycine) homopolymer in water decreases with increasing polymer molecular weight.^31,32^ During sample preparation, thin film hydration of the A_153_M_127_D_10_ polymer at 5 wt % concentration produced a visually clear solution initially, which was found to turn cloudy over time (∼2 days) upon stirring under 300 rpm at 20 °C. Time-dependent atomic force microscopy (AFM) measurements confirm the slow formation of nonspherical, elongated nanostructures (Figure S25). Figure 6a shows the representative cryo-TEM results for the 1 wt % A_153_M_127_D_10_ aqueous solution after stirred for ∼12 h and vitrified at 20 °C. As seen, A_153_M_127_D_10_ bearing a relatively large A block forms elongated fiber-like structures with several hundreds of nm in length and an average diameter of ∼10 nm, along with spherical particles surrounded by or intercalated in between. After prolonged aging at 20 °C under stirring, more fiber-like structures were formed. Interestingly, these nanofibers are not fully dispersed and randomly oriented under cryo-TEM observation but form certain aggregated structures where the nanofibers are partially aligned with an interparticle distance of ∼30 nm (Figure S26). The SANS profile of the 1 wt % A_153_M_127_D_10_ in D_2_O measured at 20 °C (*T* < *T*_gel_) after prolonged stirring shows a strong intensity upturn at low *q* region and a clear shoulder peak near *q* = 0.023 Å^–1^ (corresponding to a *d*-spacing of 27.3 nm) (Figure 6b), suggesting a preferred spacing and distinct interparticle interactions of the fibers, consistent with the cryo-TEM observation.

Remarkably, the WAXS result for the solution sample (inset of Figure 6b) reveals the absence of the characteristic (001) peak for the molecular packing of D block, indicating that the crystallization of D is suppressed due to the steric barrier provided by the large A_153_M_127_ segments. Hence, the formation of elongated nanofibers is not due to the liquid crystalline packing of the D block. Instead, there is a sharp diffraction peak located at *q* = 0.77 Å^–1^, giving a *d*-spacing of 0.82 nm. Since the M block is soluble in water, this diffraction peak can only be attributed to the crystallization of thermoresponsive A_153_ block. Previous study by Schlaad et al. has shown that the poly(*N*-allyl glycine) (A) homopolymer can crystallize after prolonged annealing at temperature above its cloud point in a 0.5 wt % aqueous solution, resulting in the formation of crystalline microparticles.^32^ Based on the theoretical molecular dimensions, the observed *d*-spacing of 0.82 nm is tentatively attributed to the interchain distance of A block separated by the *N*-allyl side chains in the crystalline lattice (Figure 6d). Hence, we speculate that the formation elongated nanofibers is driven by the crystallization of A_153_ block in aqueous solution. A full investigation on the molecular packing and phase transition of poly(*N*-allyl glycine) will be the subject of future experiments.

The above-mentioned results show that when the A block is sufficiently long, the AMD polymer chains can slowly aggregate in solution at 20 °C, that is, a temperature very close to *T*_gel_, forming elongated fiber-like A domains that are interconnected by the bridging M chains and spherical hydrophobic D domains (Figure 6d). Upon increasing temperature, the A blocks began to dehydrate and reorganize, forming macroscopic hydrogel networks presumably by generating more interconnected fiber-like domains (Figure 6d). This gives rise to the emergence of well-defined dual scattering peaks in SANS at *T* ≥ 25 °C (Figure 6c). Interestingly, unlike A_43_M_92_D_9_ and A_101_M_111_D_21_, there is no merging or broadening of the two scattering peaks for A_153_M_127_D_10_ upon further increasing temperature up to 60 °C (Figure 6c). We postulate that once the hydrogel network is formed, the interconnected crystalline nanofibers composed of A blocks cannot undergo fusion and spatial rearrangement within the two-compartment network due to the large free energy barrier imposed by crystallization.

## Conclusions

In this study, we synthesized a series of thermoresponsive triblock terpolypeptoids, poly(*N*-allyl glycine)-*b*-poly(*N*-methyl glycine)-*b*-poly(*N*-decyl glycine) (AMD), with varied chain lengths of the LCST-type A blocks and hydrophobic D blocks. To facilitate a detailed structural analysis, we also prepared partially deuterated counterparts, poly(*N*-allyl glycine)-*b*-poly(*N*-methyl glycine)-*b*-poly(*N*-decyl-*d*_21_ glycine) (AMdD), for contrast-matching SANS experiments. The structural evolution of AMD during the sol-to-gel transition was investigated using contrast-matching SANS in conjunction with X-ray scattering and microscopy techniques. At 20 °C, these AMD triblock terpolypeptoids self-assembled into either spherical or elongated micelles, depending on the relative chain lengths of the A and D blocks. Specifically, A_100_M_101_D_10_ forms spherical micelles with an amorphous D core. Upon heating above *T*_gel_, these micelles transform into a two-compartment micellar network composed of dehydrated A domains and hydrophobic D domains. Increasing temperature further above *T*_gel_ has led to further dehydration of A block and structural reorganization of two different domains, forming a better-defined binary micellar network at higher temperatures.

Interestingly, we found that the initial micellar morphology of the AMD solution significantly influences the temperature-induced structural evolution of the hydrogel network. With longer D blocks and shorter A blocks, such as A_101_M_107_D_21_ and A_43_M_92_D_9_, elongated AMD micelles were formed initially, where the D blocks are stacked into a “sanidic” LC mesophase in the micellar core. The elongated AMD micelles with relatively high aggregation number facilitate the immediate association of the elongated micelles into ordered assemblies upon A block dehydration. As the temperature further increases, the A blocks continue to dehydrate and collapse, causing both the A and D domains to rearrange within the two-compartment network. By contrast, when the A block is sufficiently long, such as in A_153_M_127_D_10_, the A blocks can crystallize to form fiber-like domains due to temperature-induced dehydration, resulting in the formation of structurally stable two-compartment networks that are more resistant to temperature perturbation. These insights into the relationship between block composition, micellar morphology, and hydrogel network structure provide valuable guidelines for the rational design of thermoresponsive triblock terpolypeptoid and other terpolymer hydrogels with tunable network structures and stimuli–responsive behaviors.

## Supporting Information Available

Synthetic procedures for *N*-decyl-*d*_21_-amine and partially deuterated decyl-*d*_21_-NCA monomer and its precursors, theoretical scattering models for polymer micelles and binary spheres with sticky hard sphere interaction, synthetic schemes of *N*-decyl-*d*_21_-amine, the decyl-*d*_21_-NCA monomer and AMD/AMdD triblock terpolypeptoids, ^1^H NMR and ^2^H NMR spectra of different chemical compounds, monomers and polymers, SEC-DRI chromatograms of different polymers, individual scattering contributions of SANS model fitting, contrast factor analysis, a comparison between eqs 2 and 3 for model fitting, additional cryo-TEM images, AFM images, SAXS/WAXS and SANS profiles for AMD solutions and hydrogels (PDF)
