# Supplementary material for: Effect of Micellar Morphology on the Temperature-Induced Structural Evolution of ABC Polypeptoid Triblock Terpolymers into Two-Compartment Hydrogel Network
Source: Macromolecules. 2024 Jun 28;57(14):6449–64. doi: 10.1021/acs.macromol.4c00162 (PMC11270984; doi:10.1021/acs.macromol.4c00162)
Supplement: Supplementary file 1 — ma4c00162_si_001.pdf [file ma4c00162_si_001.pdf]

# Supporting Information

## Effect of Micellar Morphology on the Temperature-Induced Structural Evolution of ABC Polypeptoid Triblock Terpolymers into Two-Compartment Hydrogel Network

*Naisheng Jiang,[1,2 †*] Tianyi Yu,[2, †] Meng Zhang,[2] Bailee N. Barrett,[2] Haofeng Sun,[1] Jun Wang,[1] Ying Luo,[1] Garrett L. Sternhagen,[2] Sunting Xuan,[2] Guangcui Yuan,[3] Elizabeth G. Kelley,[3] Shuo Qian,[4] Peter V. Bonnesen,[5] Kunlun Hong,[5] Dongcui Li[6] and Donghui Zhang[2*]*

[1.] Key Laboratory of Advanced Materials and Devices for Post-Moore Chips, Ministry of Education, School of Materials Science and Engineering, University of Science and Technology Beijing, Beijing 100083, China

[2.] Department of Chemistry and Macromolecular Studies Group, Louisiana State University, Baton Rouge, Louisiana 70803, United States

[3.] NIST Center for Neutron Research, National Institute of Standards and Technology, Gaithersburg, Maryland 20899, United States

[4.] Neutron Scattering Division and Second Target Station, Oak Ridge National Laboratory, Oak Ridge, Tennessee 37831, United States

[5.] Center for Nanophase Materials Sciences, Oak Ridge National Laboratory, Oak Ridge, Tennessee 37831, United States

[6.] Hua An Tang Biotech Group Co., Ltd., Guangzhou 511434, China

*Corresponding to*: dhzhang@lsu.edu and naishengjiang@ustb.edu.cn

[†]These authors contributed equally.

**Materials and Methods for Synthesis of *N*-Decyl-$d_{21}$-Amine (Scheme S1, conducted at CNMS)**

All reagents were used as received from the suppliers without further purification unless otherwise noted. Decanoic-$d_{19}$ acid (Lot X-383; stated 98.6 atom %D) was purchased from CDN Isotopes, Quebec, Canada. Lithium aluminum deuteride (98 atom %D, 90 % stated chemical purity) was obtained from the ISOTEC® Stable Isotopes division of MilliporeSigma. Proton and carbon Nuclear Magnetic Resonance spectra were obtained on a Varian VNMRS 500 NMR spectrometer at the Center for Nanophase Materials Sciences, operating at 499.715 MHz for proton, and were recorded at room temperature in $CDCl_3$ (7.27 ppm $^1$H reference and 77.23 ppm $^{13}$C reference). Carbon NMR spectra were obtained using inverse-gated decoupling with a recycle delay of 20 s.

**Scheme S1. Synthesis of the *N*-decyl-$d_{21}$-amine**

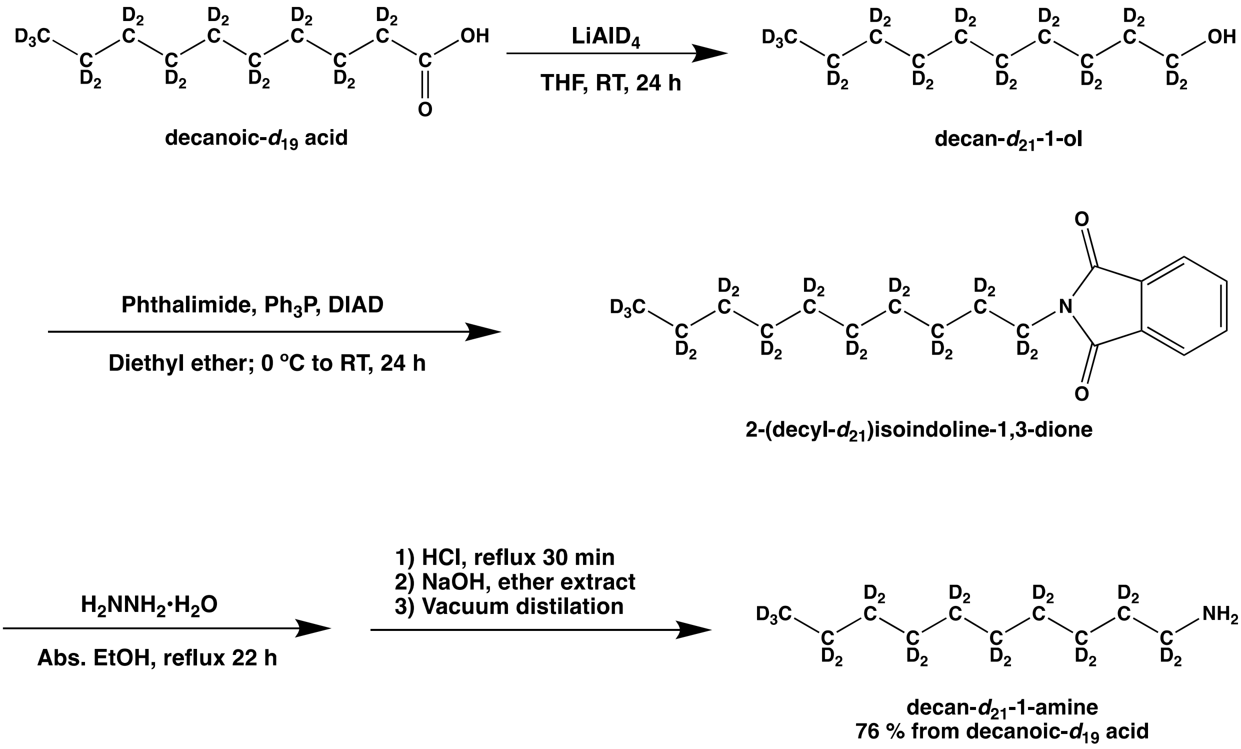


**Synthesis of Decan-$d_{21}$-1-ol.** To a stirred solution of lithium aluminum deuteride (LAD, 6.654 g, 0.158 mol) suspended in dry THF (500 mL) was added dropwise a solution of decanoic-$d_{19}$ acid (19.91 g, 0.104 mol) dissolved in 225 mL dry THF over the course of 2.5 h, at room temperature under nitrogen. The suspension was stirred an additional 24 h, then chilled in an ice bath, and deionized water (65 mL) was added dropwise over 1 h to quench the reaction. The mixture was transferred to a 2-L round bottom flask, and 1N HCl was added in portions until the pH was ≤ 2 (ca. 600 mL). Most of the

THF was then removed by rotary evaporation at 45 ºC. The remaining suspension was diluted with an additional 300 mL water, then extracted with dichloromethane (400 mL, then 3 × 350 mL). The combined dichloromethane extracts were dried ($Na_2SO_4$) and the volatiles removed by rotary evaporation to afford the crude product as a colorless liquid (19.26 g) that was used in the next step without further purification. $^{13}C\{^1H\}$ NMR ($CDCl_3$): δ 62.3 (p, $J_{CD}$ = 21.5 Hz, C1), 31.7 (p, $J_{CD}$ = 19.1 Hz, C2), 30.7 (p, $J_{CD}$ = 18.6 Hz, C8), 28.5 (four overlapping pentets, $J_{CD}$ average = 18.2 Hz, C4-C7), 24.6 (p, $J_{CD}$ = 19.1 Hz, C3), 21.5 (p, $J_{CD}$ = 19.1 Hz, C9), 13.1 (septet, $J_{CD}$ = 18.9 Hz, C10). Integration revealed the crude product to be a mixture of the alcohol (18.48 g, 0.103 mol, 99%) and residual tetrahydrofuran (ca. 0.78 g).

**Synthesis of 2-(decyl-*d*21)isoindoline-1,3-dione.** The Mitsunobu[S1] reaction was conducted in a manner similar to that previously described.[S2] The crude decan-$d_{21}$-1-ol product from the previous reaction (ca. 0.103 mol) was dissolved in dry ethyl ether (100 mL), and combined with recrystallized triphenyl phosphine (27.02 g, 0.103 mol), and phthalimide (99%, 15.30 g, nominally 0.103 mol), in a 500-mL round bottom flask under nitrogen. The flask was fitted with a 125-mL pressure-equalizing addition funnel, containing diisopropylazodicarboxylate (DIAD, 94%, 22.2 g, nominally 0.103 mol) dissolved in dry ethyl ether (35 mL). Under nitrogen flow, the flask was cooled in an ice-water bath, and the DIAD solution added dropwise over a period of 45 min. The ice bath was then removed, and the slurry stirred at room temperature for 24 h. The slurry was then filtered and the precipitate washed portionwise with ethyl ether until the precipitate cake was white and the filtrate colorless (4 x 15 mL). The solvent was removed from the filtrate by rotary evaporation to afford as a thick yellow-orange oil, which solidifies upon standing. The solid (42.59 g) is a mixture of mostly the product, triphenyl phosphine oxide, and diisopropyl hydrazidodicarboxylate, and was used in the next step without further purification. $^1H$ NMR ($CDCl_3$): δ 7.82 (m, 2H, Ar), 7.69 (m, 2H, Ar). $^{13}C\{^1H\}$ NMR ($CDCl_3$): δ 168.7 (CO), 134.0 (Ar), 132.4 (Ar), 123.3 (Ar), 37.6 (p, $J_{CD}$ = 21.5 Hz, C1-N), 30.6 (p, $J_{CD}$ = 19.1 Hz, C8), 28.2 (four overlapping pentets, $J_{CD}$ average = 18.2 Hz, C4-C7), 27.6 (p, $J_{CD}$ = 19.0 Hz, C2), 25.8 (p, $J_{CD}$ = 18.6 Hz, C3), 21.5 (p, $J_{CD}$ = 18.7 Hz, C9), 13.1 (septet, $J_{CD}$ = 19.1 Hz, C10).

**Synthesis of Decan-*d*21-1-amine (*N*-decyl-*d*21-amine).** The crude 2-(decyl-$d_{21}$)isoindoline-1,3-dione product mixture was dissolved in absolute ethanol (70 mL), and an excess of hydrazine

monohydrate (7.08 g, 0.14 mol) was added dropwise under nitrogen. The solution was then refluxed with stirring under nitrogen for 22 h, during which an ivory-colored spongy mass form. At this time, 6 M HCl (42 mL) was added to the contents and heating continued for an addition 30 min. The reaction mixture was then allowed to cool to lab temperature, then further cooled in an ice bath. The suspension was then filtered, and the solids washed with cold deionized water (2 × 30 mL). The filtrate and washings were combined, and most of the ethanol removed by rotary evaporation. The concentrated slurry was then cooled in an ice bath, and 3N NaOH added until the solution was alkaline. The alkaline solution was then extracted with diethyl ether (3 × 160 mL). The combined ether extracts were dried ($Na_2SO_4$) and the volatiles removed by rotary evaporation to afford the crude product as a turbid brown liquid (28.35 g). This mixture was allowed to stand for several days to allow the triphenyl phosphine oxide and diisopropyl hydrazidodicarboxylate that was carried over to crystallize out as a 1:1 adduct. The brown supernatant liquid phase was transferred to a distilling flask, and the crystalline residue washed with several portions of pentane (3 × 5 mL). The pentane washings also transferred to the distilling flask. The bulk of the pentane was removed by rotary evaporation, then the material was fractionally distilled under vacuum, using a jacketed Vigreux column of height 18 cm. The fraction distilling at 46 - 47 ºC (at 0.8 - 0.9 mmHg) was collected into a receiver that was cooled in an ice bath, so that the distilled amine would freeze. The yield on the colorless decan-$d_{21}$-1-amine was 14.096 g (76 % yield from the starting decanoic-$d_{19}$ acid.) $^{13}C\{^1H\}$ NMR ($CDCl_3$): δ 41.5 (p, $J_{CD}$ = 20.3 Hz, C1-N), 32.8 (p, $J_{CD}$ = 19.1 Hz, C2), 30.7 (p, $J_{CD}$ = 18.7 Hz, C8), 28.3 (four overlapping pentets, $J_{CD}$ average = 18.2 Hz, C4-C7), 25.7 (p, $J_{CD}$ = 19.1 Hz, C3), 21.5 (p, $J_{CD}$ = 18.8 Hz, C9), 13.1 (septet, $J_{CD}$ = 19.1 Hz, C10).

**Additional Experimental Section**

**Synthesis of 2-(n-decyl-*d*$_{21}$-amino)acetic Acid Hydrochloride.** Glyoxylic acid monohydrate (1.168g, 12.69 mmol) was added in $CH_2Cl_2$ with stirring at 400 rpm until the solid is fully dissolved. *N*-decyl-$d_{21}$-amine (1.132g, 6.34 mmol) was added into the above solution and the reaction was stirred at room temperature overnight. The volatiles were removed under vacuum to afford a pale-yellow oil to which 4N HCl aqueous solution (12.68 ml) was added. The mixture was then refluxed at 90 ºC overnight followed by rotary evaporation to afford a faint yellow solid, which was further purified by

recrystallization using methanol/ THF (v/v = 1:8) at 4 $^{o}$ C twice to yield a white solid (Compound **1** in Scheme S2) (0.918 g, 56.1 % yield). $^{1}$H NMR (δ in DMSO, 400 MHz, ppm): 3.83 (s, -$COCH_2$-), 9.06 (s, -HNHCl). $^{2}$H NMR (δ in DMSO, 400 MHz, ppm): 0.74 ppm (t, $CD_3(CD_2)_7CD_2CD_2$-); 1.13 ppm (m, $CD_3(CD_2)_7CD_2CD_2$-).

**Synthesis of 2-(*N,N*-tert-butoxycarbonyl-n-decyl-$d_{21}$-amino)acetic Acid.** Compound **1** (Scheme S2) (0.918 g, 3.55 mmol), di-*tert*-butyl dicarbonate (1.937 g, 8.88 mmol) and triethylamine (2.66 ml, 17.75 mmol) were dissolved in DI water (17.75 ml) and stirred at 25 ºC for 18 h. The reaction mixture was extracted with hexanes (3 × 10 ml) to remove excess di-*tert*-butyl dicarbonate. Note that an emulsion layer was formed between the aqueous and hexanes phases in the presence of amphiphilic Compound **1**, which was not collected, thus resulting in the partial loss of the products. The aqueous phase was isolated and acidified with 4N HCl till pH = 4 at 0 ºC. The aqueous solution was further extracted with ethyl acetate (3 × 10 ml). The separated organic layer was washed with brine and dried over anhydrous $MgSO_4$. After filtration, the volatiles were removed under vacuum to afford the desired product as a colorless oil (Compound **2** in Scheme S2) (0.360 g, 30.2 % yield). $^{1}$H NMR (δ in $CDCl_3$, 400 MHz, ppm): 3.96 and 3.87 (s, -$COCH_2$-); 1.46 and 1.42 (s, -$(CH_3)_3$). $^{2}$H NMR (δ in $CDCl_3$ 400 MHz, ppm): 0.76 ppm (t, $CD_3(CD_2)_7CD_2CD_2$-); 1.17 ppm (m, $CD_3(CD_2)_7CD_2CD_2$-); 1.40 (m, $CD_3(CD_2)_7CD_2CD_2$-), 3.15 (m, $CD_3(CD_2)_7CD_2CD_2$-).

**Synthesis of Deuterated *N*-decyl-$d_{21}$ Substituted *N*-carboxyanhydride (Decyl-$d_{21}$-NCA) Monomer.** Compound **2** (Scheme S2) (0.360 g, 1.07 mmol) was dissolved in anhydrous $CH_2Cl_2$ (5.4 ml, 0.2 M) under nitrogen at 0 ºC, and phosphorus trichloride (78 µl, 0.89 mmol) was added dropwise with stirring. Under nitrogen atmosphere, the reaction mixture was stirred at 0 ºC for 1 h before warmed to room temperature for additional 3 h. The reaction mixture was concentrated and purified by passing through a silica plug. The filtrate was dried under vacuum to afford a white oil, which was further extracted using anhydrous $CH_2Cl_2$ (3× 30 ml). The organic extract was stirred over $CaH_2$ (40 mg, 0.95 mmol) for 20 min to remove any acidic impurities, filtered and evaporated under vacuum to afford a solid, which was further purified by recrystallization twice with $CH_2Cl_2$/hexanes (v/v = 1: 10) at -20 ºC to afford a white solid (Monomer **M** in Scheme S2) (62.7 mg, 22.3 % yield). $^{1}$H NMR (δ in $CDCl_3$, 400 MHz, ppm): 4.11 (s, -$COCH_2$-); $^{2}$H NMR (δ in $CDCl_3$ 400 MHz, ppm): 0.80 ppm (t,

$CD_3(CD_2)_7CD_2CD_2$-); 1.17 ppm (m, $CD_3(CD_2)_7CD_2CD_2$-); 1.51 (m, $CD_3(CD_2)_7CD_2CD_2$-), 3.34 (t, $CD_3(CD_2)_7CD_2CD_2$-).

**Theoretical Scattering Model for Polymer Micelles with a Spherical Core.** According to the early description by Pedersen and coworkers,[S3, 4] the scattering form factor of a single micelle with a homogeneous core surrounded by Gaussian corona chains contains four different terms: the self-correlation term of the core, the self-correlation term of the corona chains, the cross term between the core and corona chains, and the cross term between different chains in the corona. The equation can be written as:

$$P_{\mathrm{mic}}(q) = N_{\mathrm{agg}}^{2}\beta_{\mathrm{core}}^{2}P_{\mathrm{core}}(q) + N_{\mathrm{agg}}\beta_{\mathrm{corona}}^{2}P_{\mathrm{corona}}(q) + 2N_{\mathrm{agg}}^{2}\beta_{\mathrm{core}}\beta_{\mathrm{corona}}S_{\mathrm{core\text{-}corona}}(q) + N_{\mathrm{agg}}(N_{\mathrm{agg}} - 1)\beta_{\mathrm{corona}}^{2}S_{\mathrm{corona\text{-}corona}}(q) \qquad \text{(eq. S1)}$$

In this equation, $q$ is the magnitude of the scattering vector, $N_{agg}$ is the aggregation number of the micelle, $\beta_{core}$ and $\beta_{corona}$ are the total excess scattering lengths of the core block and the corona block, respectively. Assuming the core is completely dry, $\beta_{core}$ and $\beta_{corona}$ are defined as: $\beta_{core} = (\rho_{core} - \rho_{solvent})V_{core}$ and $\beta_{corona} = (\rho_{corona} - \rho_{solvent})V_{corona}$, where $\rho_{core}$, $\rho_{corona}$ and $\rho_{solvent}$ are the scattering length densities of the core-forming D block, corona-forming A and M blocks and solvent ($D_2O$), respectively; $V_{core}$ and $V_{corona}$ are the molecular volumes of a single D block in the core and an A-*b*-M chain in the corona, respectively. Based on the reported bulk densities of poly(*N*-allyl glycine), poly(*N*-methyl glycine) and poly(*N*-decyl glycine),[S5-S8] the $\rho_{core}$ and $\rho_{corona}$ values can be obtained via the equation: $\rho = (\Sigma_i n_i b_i)/V$, where $b_i$ is the bound coherent scattering length of atomic species $i$, $n_i$ is the number of atoms of $i$ per chain, $V$ is the molecular volume of a single chain that is defined by $V = (M_0/\rho_{mass})/N_A$, where $M_0$ is the molar mass of the chain, $\rho_{mass}$ is the mass density of the polymeric material, and $N_A$ is Avogadro's number.

For a spherical core with a core radius of $R_c$, the aggregation number ($N_{agg}$) is expressed by $N_{agg} = 4\pi R_c^3/3V_{core}$., assuming the core contains no solvent. The self-correlation term of the core, $P_{core}(q)$, in eq. S1 can be written as:

$$P_{\mathrm{core}}(q) = \Phi^{2}(qR_{\mathrm{c}}) \qquad \text{(eq. S2)}$$

where

$$\Phi^2(qR_c) = \frac{3[\sin(qR_c) - qR_c\cos(qR_c)]}{(qR_c)^3} \qquad \text{(eq. S3)}$$

where $R_c$ is the radius of the spherical core.

For the Gaussian chains with a radius of gyration of $R_g$ in the corona, the self-correlation term of the corona chains, $P_{corona}(q)$, is therefore given by the Debye function:[S9]

$$P_{corona}(q) = \frac{2[\exp(-q^2R_g{}^2) + q^2R_g{}^2 - 1]}{q^4R_g{}^4} \qquad \text{(eq. S4)}$$

According to the description, the Gaussian chains are uniformly distributed at a distance $d_{int}R_g$ away from the surface of the disk core, where $d_{int}$ is close to unity as to mimic non-penetration of the corona chains into the core region.[S3, S4]

The interference cross term between the spherical core and the corona chains, $S_{core\text{-}corona}(q)$, and the interference cross term between the corona chains, $S_{corona\text{-}corona}(q)$, are then written as:

$$S_{core\text{-}corona}(q) = \Phi(qR_c)\frac{1 - \exp(-q^2R_g{}^2)}{q^2R_g{}^2}\frac{\sin[q(R_c + d_{int}R_g)]}{q(R_c + d_{int}R_g)} \qquad \text{(eq. S5)}$$

$$S_{corona\text{-}corona}(q) = \frac{[1 - \exp(-q^2R_g{}^2)]^2}{q^4R_g{}^4}\left[\frac{\sin[q(R_c + d_{int}R_g)]}{q(R_c + d_{int}R_g)}\right]^2 \qquad \text{(eq. S6)}$$

The form factor of the polymer micelles with a spherical core can be then obtained by inserting the above terms into eq. S1.

The interparticle structure factor, S($q$), which describes the positional correlation of polymer micelles relative to one another, is included in the SANS analysis when interparticle interactions contribute significantly in the scattering profile. For simplicity, the S($q$) is described by a hard-sphere interaction that considers short-range repulsive potentials between particles, which depends on the hard-sphere interaction radius ($R_{HS}$) and hard-sphere volume fraction ($\eta_{HS}$).[S10-S13] The detailed analytical expression of S($q$) has been described elsewhere.[S12, S13] Note that $R_{HS}$ can be described as $R_{HS} = R_c + \Delta R_{HS}$, where $\Delta R_{HS}$ is related to the width of the corona region and is approximately $2R_g$.[S10, S11] The final expression for the scattering intensity using the decoupling approximation is then given by:

$$I(q) = n(P_{mic}(q) + A_{mic}(q)^2(S(q) - 1)) + I_{inc} \qquad \text{(eq. S7)}$$

where $n$ is the number density of micelles in the system, $A_{mic}(q)$ is the form factor amplitude of the radial scattering length distribution of the micelle, and $I_{inc}$ is a $q$-independent term accountable the

incoherent scattering of solvent and hydrogen atoms in the sample. In case where there is little to no interparticle interaction observed, i.e., $S(q) \approx 1$, the expression for $I(q)$ is then given by:

$$I(q) = nP_{\mathrm{mic}}(q) + I_{\mathrm{inc}} \qquad \text{(eq. S8)}$$

The polydispersity of the core radius can also be considered during the model fitting, assuming a Gaussian number distribution for the core radii, where the Gaussian distribution function is given by:

$$G(R_c) = \frac{1}{\sqrt{2\pi}\sigma_{R_c}} \exp\left[\frac{-(R_c - \langle R_c \rangle)^2}{2\sigma_{R_c}{}^2}\right] \qquad \text{(eq. S9)}$$

where $\langle R_c \rangle$ is the mean radius of the core and $\sigma_{R_c}$ is the standard deviation of the distribution truncated at $R_c = 0$.

**Theoretical Scattering Model for Polymer Micelles with a Rod-Shaped Core.** For polymer micelles with a rod-shaped core, the form factor has a similar description to the spherical micelle model described above, except for the difference in the geometrical shape of the micellar core. For a micelle with a rod-shaped core, the self-correlation term of the core, $P_{\mathrm{core}}(q)$, is given by:[S4]

$$P_{\mathrm{core}}(q) = \int_0^{\pi/2} \Psi^2(q, R_c, L_c, \alpha) \sin\alpha \, d\alpha \qquad \text{(eq. S10)}$$

where

$$\Psi(q, R_c, L_c, \alpha) = \frac{2J_1(qR_c \sin\alpha)}{qR_c \sin\alpha} \frac{\sin\left(\frac{1}{2} qL_c \cos\alpha\right)}{\frac{1}{2} qL_c \cos\alpha} \qquad \text{(eq. S11)}$$

where $\alpha$ is the angle between $q$ and the axis of the cylinder parallel to $L_c$, and $J_1(x)$ is the first order Bessel function of the first kind.

When $L_c >> R_c + d_{\mathrm{int}}R_g$, $P_{\mathrm{core}}(q)$ can be simplified as:

$$P_{\mathrm{core}}(q) = \left[\frac{2J_1(qR_c)}{qR_c}\right]^2 \left[\frac{2\mathrm{Si}(qL_c)}{qL_c} - \frac{4\sin^2(qL_c/2)}{q^2 L_c{}^2}\right] \qquad \text{(eq. S12)}$$

where

$$\mathrm{Si}(x) = \int_0^x t^{-1} \sin t \, dt \qquad \text{(eq. S13)}$$

The self-correlation term of the corona chains, $P_{\mathrm{corona}}(q)$, maintains the same expression as described in eq. S4. In case of $L_c >> R_c + d_{\mathrm{int}}R_g$, the two cross terms, $S_{\mathrm{core\text{-}corona}}(q)$ and $S_{\mathrm{corona\text{-}corona}}(q)$, can be approximated by:

$$S_{\text{core-corona}}(q) = \frac{1-\exp(-q^2 R_g{}^2)}{q^2 R_g{}^2}\left[\frac{2\mathrm{J}_1(qR_c)}{qR_c}\right]\mathrm{J}_0\left[q\left(R_c + d_{\text{int}} R_g\right)\right]\left[\frac{2\mathrm{Si}(qL_c)}{qL_c} - \frac{4\sin^2(qL_c/2)}{q^2 L_c{}^2}\right]$$

(eq. S14)

$$S_{\text{corona-corona}}(q) = \frac{\left[1-\exp(-q^2 R_g{}^2)\right]^2}{q^4 R_g{}^4}\mathrm{J}_0{}^2\left[q\left(R_c + d_{\text{int}} R_g\right)\right]\left[\frac{2\mathrm{Si}(qL_c)}{qL_c} - \frac{4\sin^2(qL_c/2)}{q^2 L_c{}^2}\right] \quad \text{(eq. S15)}$$

where $J_0(x)$ is the zeroth order Bessel function of the first kind.

Note that the polydispersity of the radius of rod-shaped core is considered during the model fitting, assuming a LogNormal distribution for the core radii, where the LogNormal distribution function is given by:

$$\mathrm{LN}(R_c) = \frac{1}{\sqrt{2\pi} R_c \sigma_{R_c}} \exp\left[\frac{-(\ln R_c - \langle R_c \rangle)^2}{2\sigma_{R_c}{}^2}\right] \quad \text{(eq. S16)}$$

where $\langle R_c \rangle$ is the mean radius of the core and $\sigma_{R_c}$ is the standard deviation of the distribution truncated at $R_c = 0$.

It is worth to mention that for polymer micelles with an elongated, rod-shaped core, quantifying intermicellar interactions via small-angle scattering can be challenging due to their anisotropic nature. Here, we applied the hard-sphere interaction model[S12, S13] using the decoupling approximation (eq. S7) for a rough estimation of the intermicellar interactions. In this case, the hard-sphere interaction radius, $R_{HS}$, serves as an "effective" parameter due to the assumption of centrosymmetric interactions, which may not be able to fully present the actual interactions of elongated micelles.[S14]

**Theoretical Scattering Model for a Binary Mixture of Hard Spheres with Sticky Hard Sphere Interaction.** For the two-compartment hydrogel network, scattering contribution from the two distinct spherical domains can be described by form factors for a binary mixture of hard spheres, while structure factors elucidate the sticky hard-sphere interactions between them. The corresponding expression for the scattering intensity is given by:

$$\mathrm{I}(q) = n_1 \mid \mathrm{A}_1(q) \mid^2 \mathrm{S}_{11}(q) + 2\sqrt{n_1 n_2}\mathrm{A}_1(q)\mathrm{A}_2(q)\mathrm{S}_{12}(q) + n_2 \mid \mathrm{A}_2(q) \mid^2 \mathrm{S}_{22}(q) \quad \text{(eq. S17)}$$

where $n_1$ and $n_2$ are number densities, $A_1(q)$ and $A_2(q)$ are the form factor amplitudes for binary hard-spheres, which are described by the total volume fraction of the polymer ($f_p$), the hard-sphere volume fractions, micellar radii, and scattering length density contrast between the two types of spheres. In the equation, $S_{11}(q)$, $S_{12}(q)$, and $S_{22}(q)$ are the sticky hard-sphere structure factors that describe the

interactions between 1-1, 1-2, and 2-2 spheres by considering both repulsive and attractive interactions.

For the hydrophobic D domain, which is anticipated to be completely dehydrated at all temperatures, its number density, $n_1$, can be described through the relationship between its volume fraction and the volume of an individual spherical domain. This relationship is given by:

$$n_1 = \frac{3 f_{\mathrm{AMD}} f_{\mathrm{D}}}{4\pi {R_{\mathrm{c,\,D}}}^3} \tag{eq. S18}$$

where $f_{\mathrm{AMD}}$ is the volume fraction corresponding to the concentration of the triblock terpolymer in aqueous solution, $f_{\mathrm{D}}$ is the volume fraction of the D block within the polymer, $R_{\mathrm{c,\,D}}$ is the radius of the spherical D domain.

For the A domain, which is thermoresponsive, its hydration state varies with temperature. At higher temperatures, the A domain undergoes dehydration but may still contain residual water. In this case, its number density, $n_2$, is described by the effective volume fraction of the A domain after accounting for the volume fraction of the retained solvent within the A domain. Therefore, $n_2$ is given by:

$$n_2 = \frac{3[f_{\mathrm{AMD}} f_{\mathrm{D}} - (1 - f_{\mathrm{AMD}})\eta_{\mathrm{water,\,A}}]}{4\pi {R_{\mathrm{c,\,A}}}^3} \tag{eq. S19}$$

where $\eta_{\mathrm{water,\,A}}$ is the fraction of water content within the A domain, $f_{\mathrm{A}}$ is the volume fraction of the A block within the polymer, $R_{\mathrm{c,\,A}}$ is the radius of the spherical A domain.

The form factor amplitude for the D and A domains, $\mathrm{A}_1(q)$ and $\mathrm{A}_2(q)$, is given by:

$$\mathrm{A}_1(q) = (\rho_1 - \rho_{\mathrm{M+water}})\ 4\pi {R_{\mathrm{c,\,D}}}^3\ \frac{[\sin(qR_{\mathrm{c,\,D}}) - qR_{\mathrm{c,\,D}}\cos(qR_{\mathrm{c,\,D}})]}{(qR_{\mathrm{c,\,D}})^3}\ \exp\left(\frac{-q^2{\omega_{\mathrm{D}}}^2}{2}\right) \tag{eq. S20}$$

$$\mathrm{A}_2(q) = (\rho_2 - \rho_{\mathrm{M+water}})\ 4\pi {R_{\mathrm{c,\,A}}}^3\ \frac{[\sin(qR_{\mathrm{c,\,A}}) - qR_{\mathrm{c,\,A}}\cos(qR_{\mathrm{c,\,A}})]}{(qR_{\mathrm{c,\,A}})^3}\ \exp\left(\frac{-q^2{\omega_{\mathrm{A}}}^2}{2}\right) \tag{eq. S21}$$

Where $\rho_1$ and $\rho_2$ are the scattering length densities (SLDs) of the D and A spherical domains, respectively. $\rho_{\mathrm{M+water}}$ is the SLD of the surrounding medium, consisting of solvated M blocks and water within the gel phase. To account for the potential smeared interface between the spherical domains and the surrounding medium, an additional term $\exp(-q^2\omega^2/2)$ has been included, where $\omega_{\mathrm{D}}$ and $\omega_{\mathrm{A}}$ are the widths of the diffused interfaces for the D and A domains, respectively.

For the D domain, $\rho_1$ is simply given by $\rho_1 = \rho_{\mathrm{D}}$, where $\rho_{\mathrm{D}}$ is the SLD of the core-forming D block.

For the A domain, $\rho_2$ is given by:

$$\rho_2 = \frac{f_{AMD} f_A \rho_A + (1 - f_{AMD})\eta_{water,\,A}\,\rho_{water}}{f_{AMD} f_A + (1 - f_{AMD})\eta_{water,\,A}} \quad \text{(eq. S22)}$$

where $\rho_A$ is the SLD of the thermoresponsive A block.

For the SLD of the surrounding medium which is consisted of solvated M block and water within the gel phase, $\rho_{M+water}$ is given by:

$$\rho_{M+water} = \frac{f_{AMD} f_M \rho_M + (1 - f_{AMD})\left(\eta_{water,\,gel} - \eta_{water,\,A}\right)\rho_{water}}{f_{AMD} f_M + (1 - f_{AMD})\left(\eta_{water,\,gel} - \eta_{water,\,A}\right)} \quad \text{(eq. S23)}$$

where $\eta_{water,\,gel}$ is the fraction of water content within the gel phase.

For the two-compartment hydrogel networks comprised of ABC-type triblock terpolymers, previous studies have demonstrated that the spherical domains of types 1 and 2 display a non-random, alternating arrangement, which is mediated by the hydrophilic mid-blocks that bridge them.[S13] Therefore, it is reasonable to employ a "sticky" hard-sphere model for the structure factor.[S15-S17] In this model, the short-range repulsion between the spheres is described by the hard-sphere diameter, which gives a minimum distance that the centers of two spheres can approach each other. On the other hand, a narrow attractive well term is introduced to account for the short-range attractions between alternating types of spheres, thus favoring 1-2 contacts over 1-1 and 2-2 contacts. For a binary mixture of hard spheres, the interaction potential as a function of the distance $r$ between the centers of two spheres is described by a square-well potential, which is given by:

$$U_{i,j}(r) = \begin{cases} \infty & 0 < r < D_{i,j} \\ -\epsilon_{i,j} & D_{i,j} < r < \mathrm{D}_{i,j} + \Delta_{i,j} \\ 0 & r > D_{i,j} + \Delta_{i,j} \end{cases} \quad \text{(eq. S24)}$$

where $D_{i,j}$ is the average hard-sphere diameter of spheres of type $i$ and $j$, which is defined as the mean of their diameters $D_i$ and $D_j$, i.e., $D_{i,j} = (D_i + D_j)/2$. Here, $\epsilon_{i,j}$ is the depth of the attractive potential well, and $\Delta_{i,j}$ is the width of the square well, which represents the range of the adhesive interaction between the $i$ and $j$ spheres. The depth $\epsilon_{i,j}$ can be expressed in terms of the "stickiness" parameter $\tau$ using the equation:

$$\tau = \exp\left(\frac{-\epsilon_{i,j}}{k_B \mathrm{T}}\right)\frac{12\Delta_{i,j}}{D_{i,j} + \Delta_{i,j}} \quad \text{(eq. S25)}$$

where $k_B$ is the Boltzmann constant and T is the temperature. By varying the depth and width of the attractive well, the "stickiness" $\tau$ can be adjusted, thereby modulating the interaction strength between the spheres. In this study, the perturbation parameter, $\Delta_{i,j}/(D_{i,j} + \Delta_{i,j})$, is fixed to 0.05 for simplicity.[S16] The interparticle structure factors $S_{11}(q)$, $S_{12}(q)$, and $S_{22}(q)$ can be then estimated as functions of the volume fractions and hard-sphere diameters (or number densities) of the two types of spheres within the gel phase, as well as the stickiness parameter $\tau$ that quantifies the strength of interaction between them.[S13]

**Scheme S2. Synthesis of the Decyl-$d_{21}$-NCA monomer**

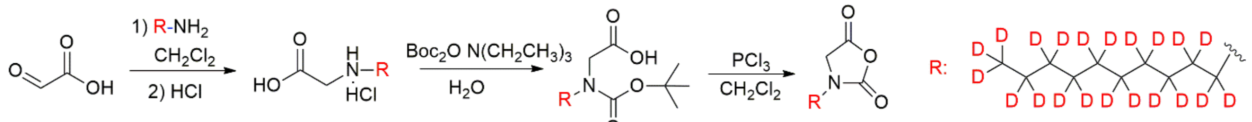


R-NCA

Compound **1** Compound **2** Monomer (M)

**Scheme S3. Synthesis of AMD or AMdD triblock terpolypeptoids**

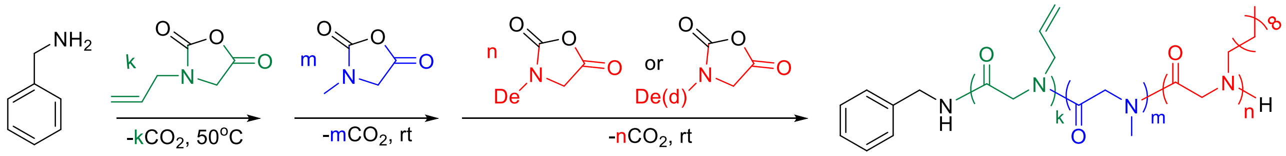

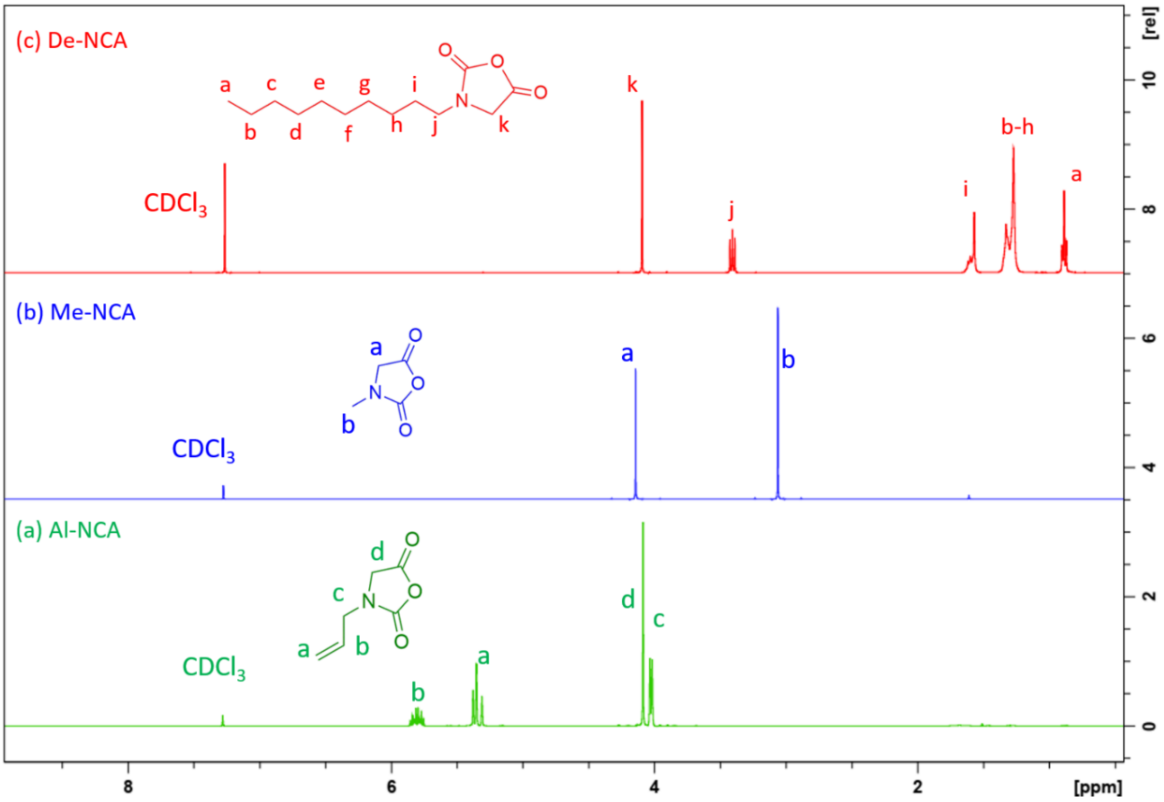


**Figure S1**. $^{1}$H NMR spectra of (a) Al-NCA, (b) Me-NCA and (c) De-NCA monomers in $CDCl_3$.

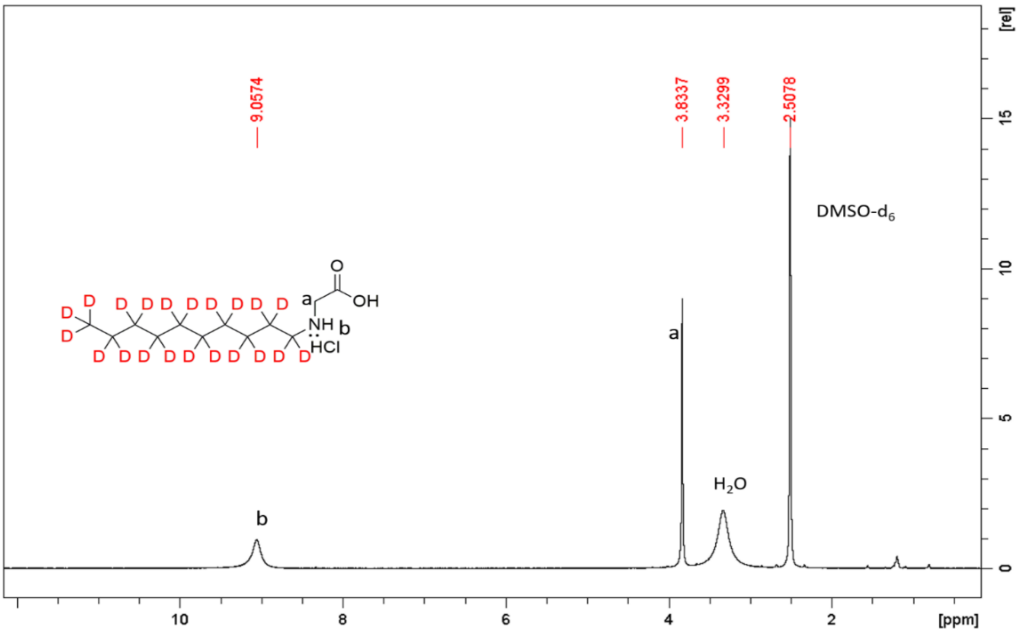


**Figure S2**. $^{1}$H NMR spectrum of 2-($n$-decyl-$d_{21}$-amino)acetic acid hydrochloride in DMSO-$d_6$.

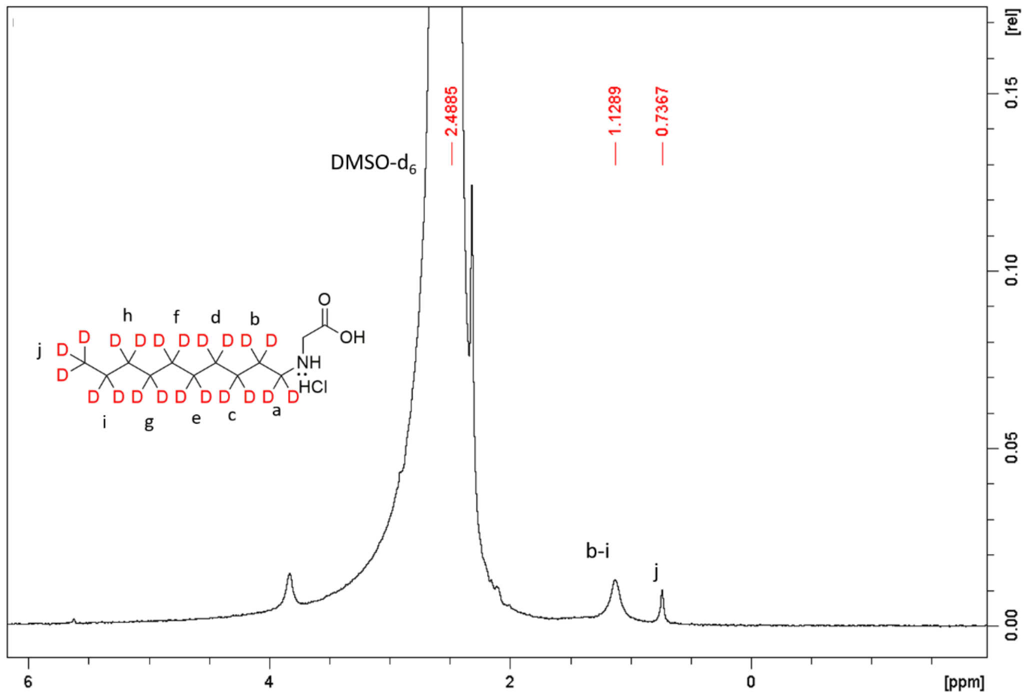


**Figure S3**. $^{2}$H NMR spectrum of 2-(*n*-decyl-$d_{21}$-amino)acetic acid hydrochloride in DMSO-$d_6$.

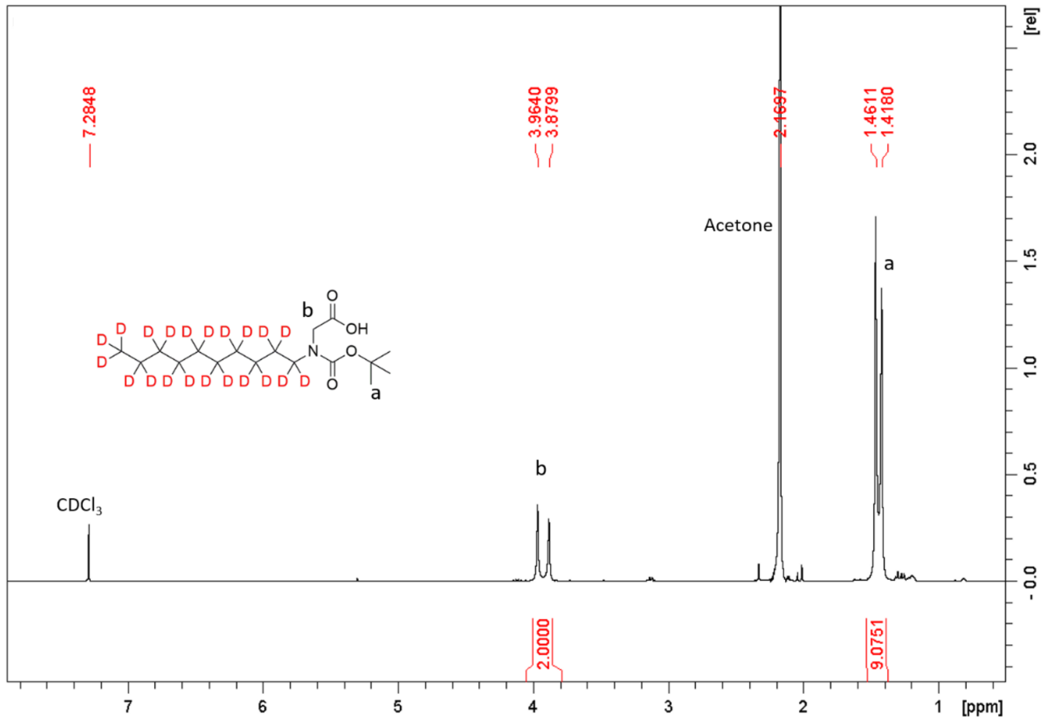


**Figure S4**. $^{1}$H NMR spectrum of 2-(*N*,*N*-*tert*-butoxycarbonyl-n-decyl-$d_{21}$-amino)acetic acid (Compound **2**) in $CDCl_3$.

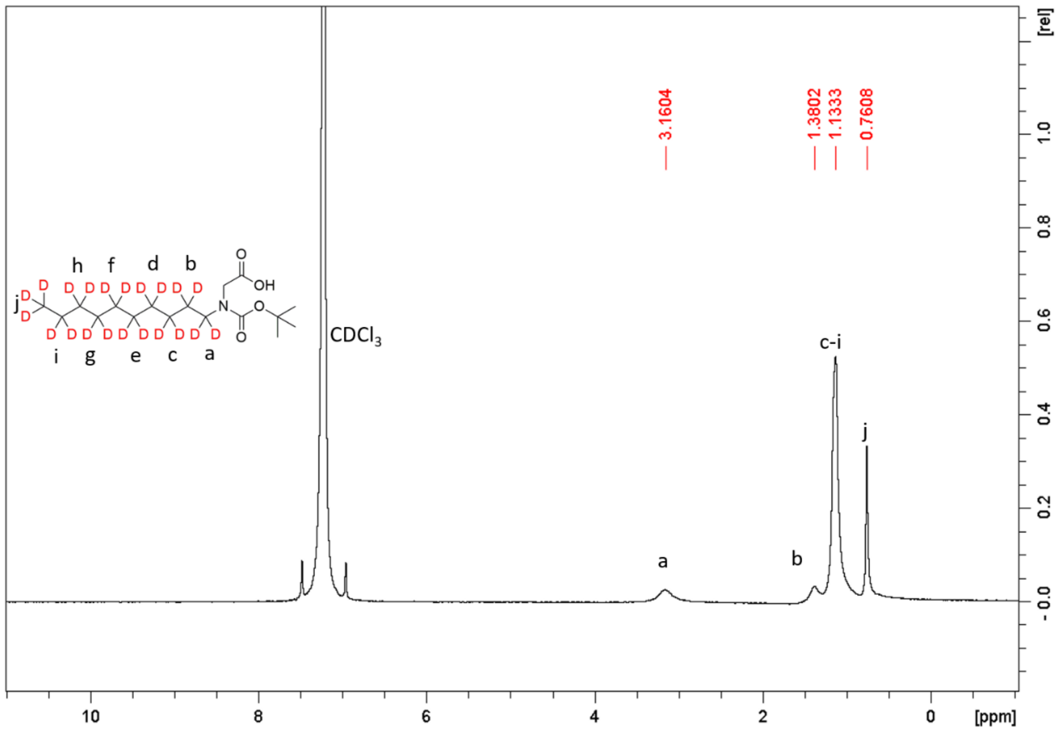


**Figure S5**. $^2$H NMR spectrum of 2-(*N*,*N*-*tert*-butoxycarbonyl-*n*-decyl-$d_{21}$-amino)acetic acid (Compound **2**) in $CDCl_3$.

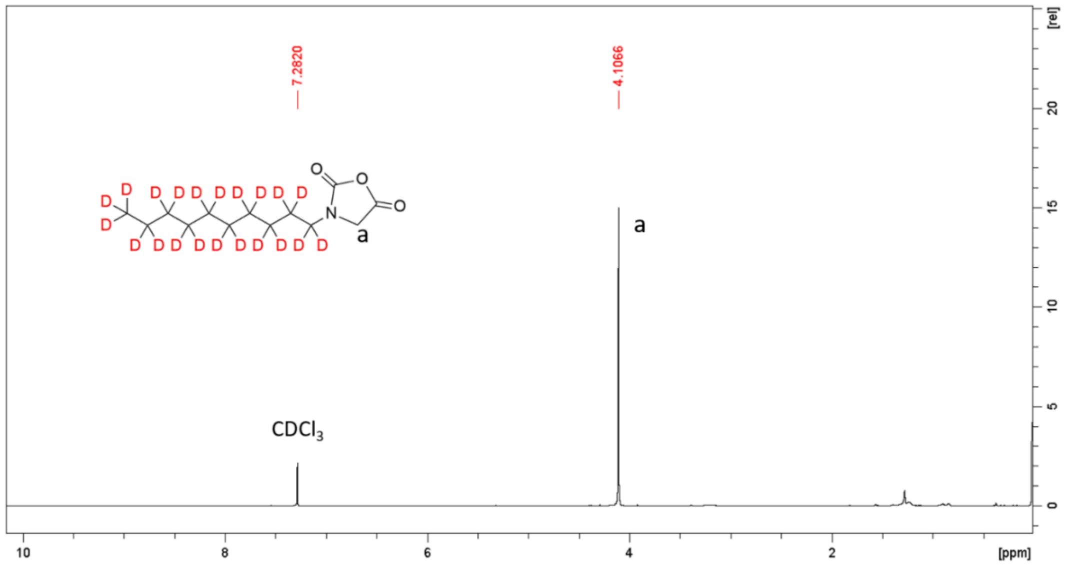


**Figure S6**. $^1$H NMR spectrum of De-$d_{21}$-NCA monomer in $CDCl_3$.

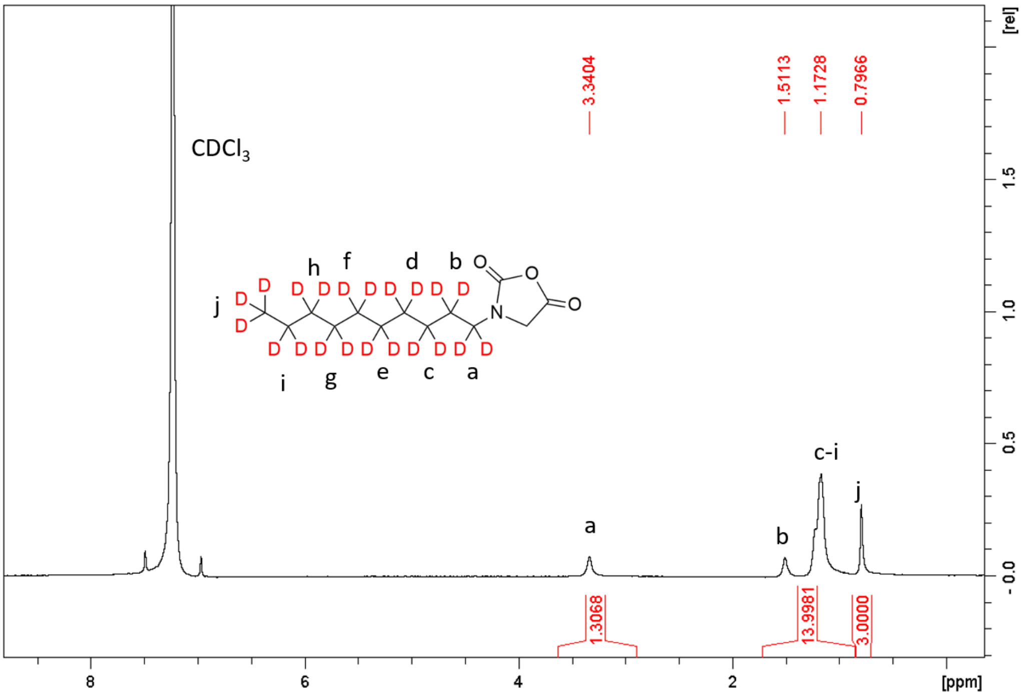


**Figure S7**. $^{2}$H-NMR spectrum of De-$d_{21}$-NCA monomer in $CDCl_3$.

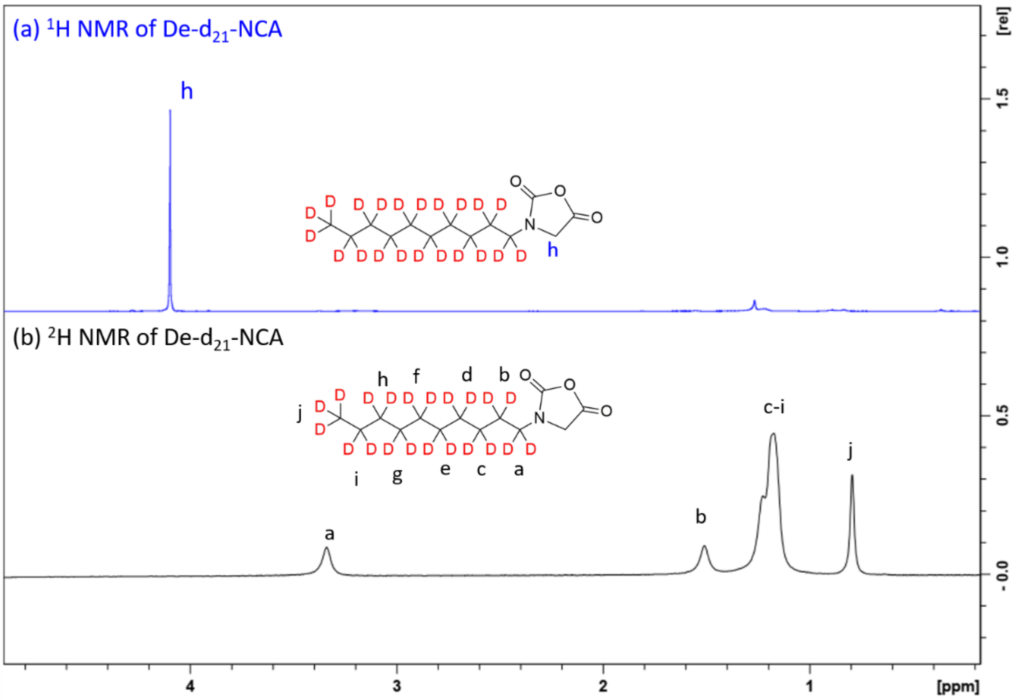


**Figure S8**. A comparison between (a)$^{1}$H-NMR and (b) $^{2}$H-NMR spectra of the De-$d_{21}$-NCA monomer in $CDCl_3$. In the $^{1}$H NMR spectrum, only the –$COCH_2$– protons appear at 4.11 ppm. The deuterated *N*-$C_{10}D_{21}$ group, invisible in the $^{1}$H NMR spectrum, is clearly observed in the $^{2}$H NMR spectrum.

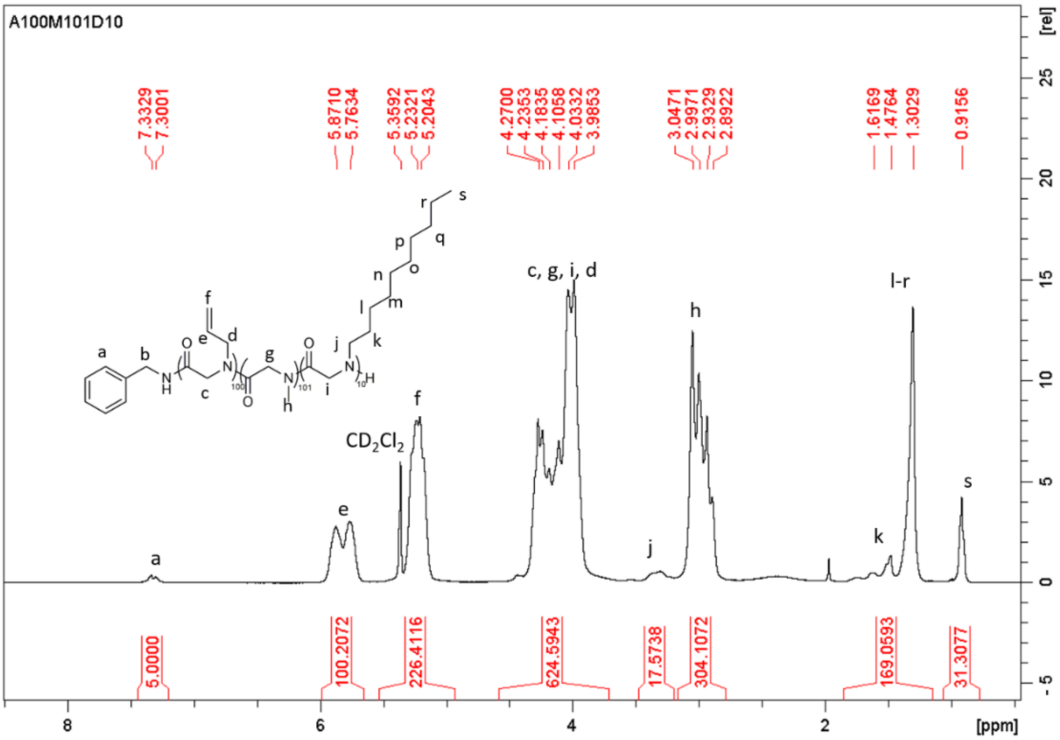


**Figure S9**. $^{1}$H NMR spectrum of $A_{100}M_{101}D_{10}$ triblock terpolymer in $CD_2Cl_2$.

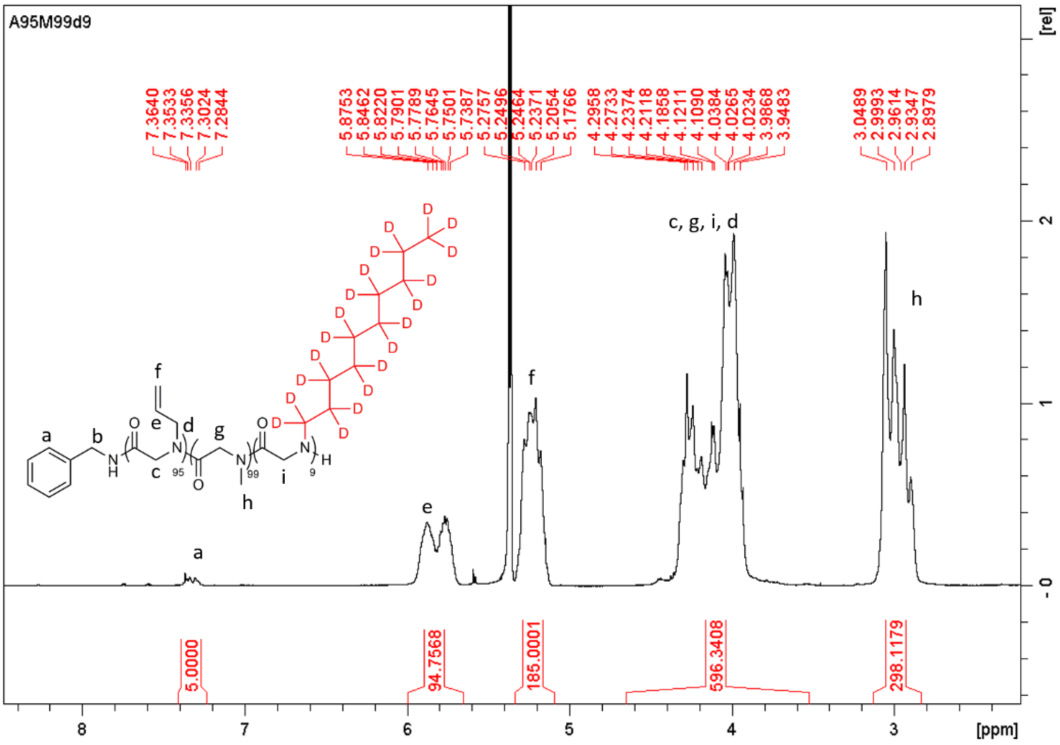


**Figure S10**. $^{1}$H NMR spectrum of $A_{95}M_{99}dD_9$ triblock terpolymer in $CD_2Cl_2$.

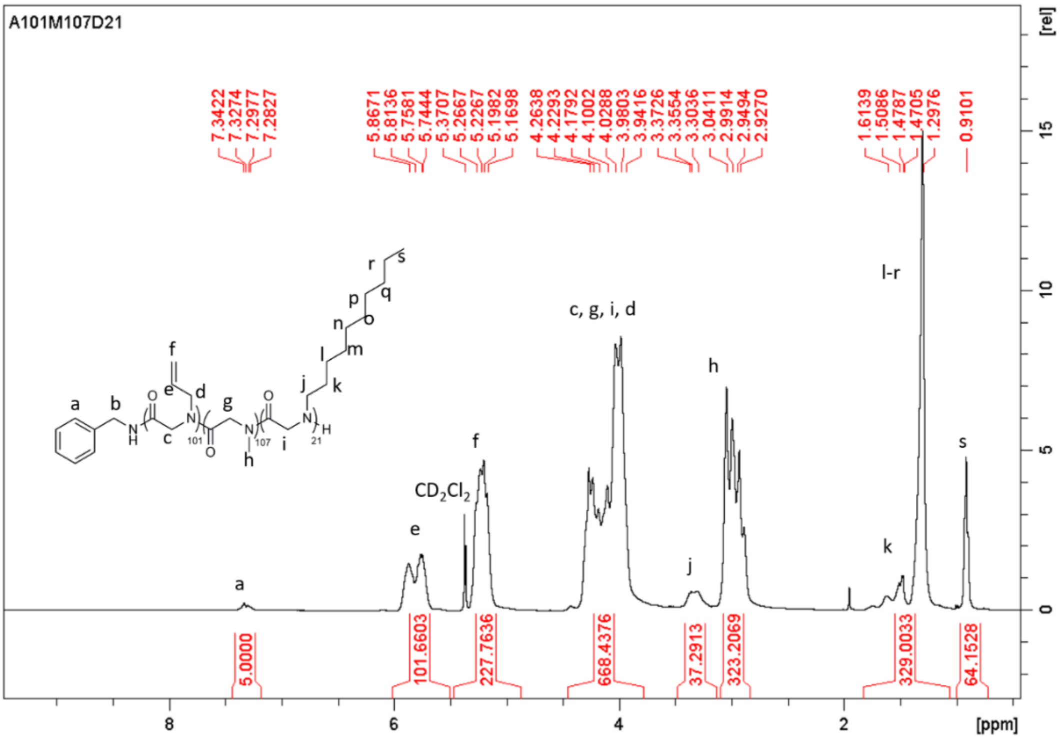


**Figure S11**. $^{1}$H NMR spectrum of $A_{101}M_{107}D_{21}$ triblock terpolymer in $CD_2Cl_2$.

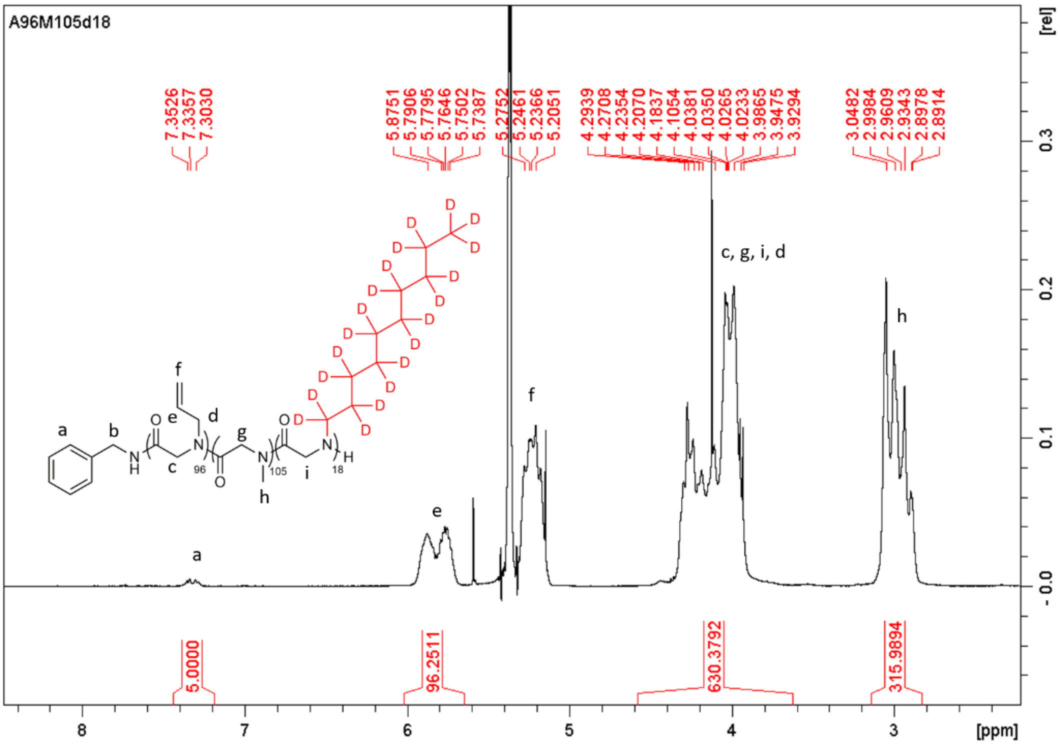


**Figure S12**. $^{1}$H NMR spectrum of $A_{96}M_{105}dD_{18}$ triblock terpolymer in $CD_2Cl_2$.

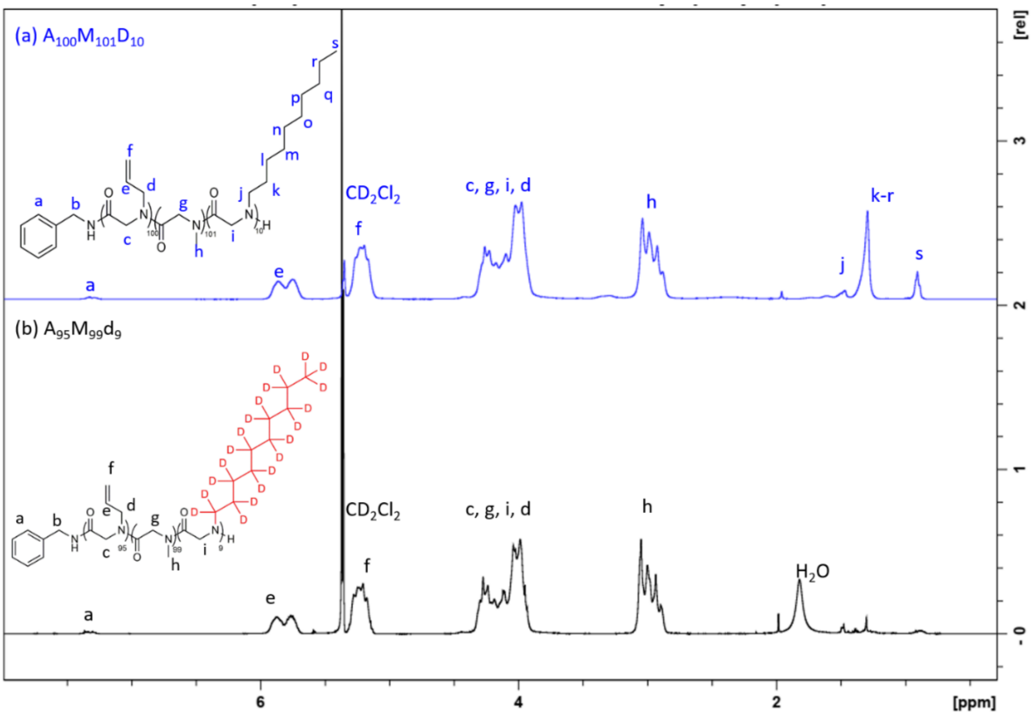


**Figure S13**. A comparison between $^{1}$H NMR spectra of (a) $A_{100}M_{101}D_{10}$ and (b) $A_{95}M_{99}dD_9$ triblock terpolymers in $CD_2Cl_2$. Note that the methods for calculating $DP_n$ (A) and $DP_n$ (M), based on the end group analysis, are the same for both AMD and AMdD terpolymers: $DP_n$ (A) = (5 × integration of –CH=) / (1 × integration of $–C_6H_5$); $DP_n$ (M) = (5 × integration of a–$CH_3$) / (3 × integration of $–C_6H_5$), where "a" refers to the methyl protons in the M block. By contrast, the method to calculate $DP_n$ (D) for AMD and $DP_n$ (dD) for AMdD are different. For AMD terpolymers, $DP_n$ (D) = (5 × integration of b–$CH_3$) / (3 × integration of $–C_6H_5$), where "b" refers to the methyl protons in the D block. For AMdD terpolymers, the deuteriums on the side chain of the dD segment are invisible in the $^{1}$H NMR spectrum, while the $–CH_2–$ on the backbone are hydrogenated, which can be used to determine the $DP_n$ (dD) in $^{1}$H NMR. Therefore, $DP_n$ (dD) = (5 × (integration of $–COCH_2N–$) / (2 × integration of $–C_6H_5$) − $DP_n$(A) − $DP_n$(M)).

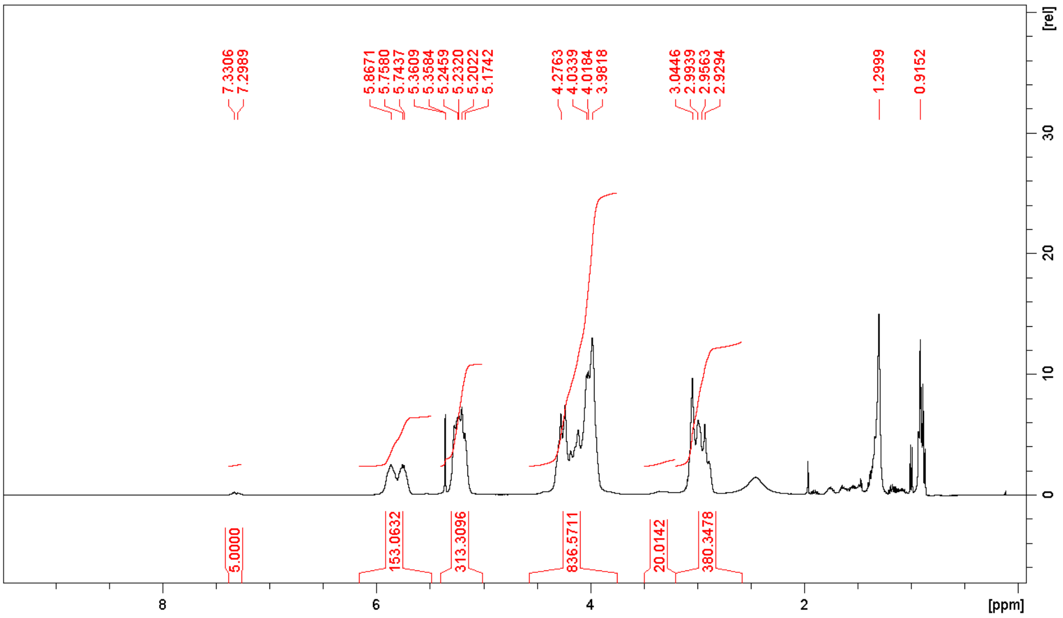


**Figure S14**. $^{1}$H NMR spectrum of $A_{153}M_{127}D_{10}$ triblock terpolymer in $CD_2Cl_2$.

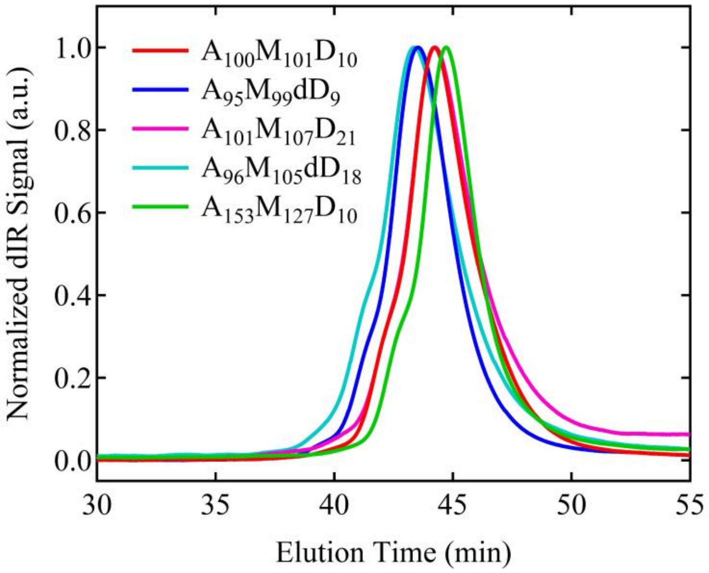


**Figure S15**. SEC-DRI chromatograms of various AMD and AMdD triblock terpolymers in DMF with 0.1 M LiBr at 25 °C.

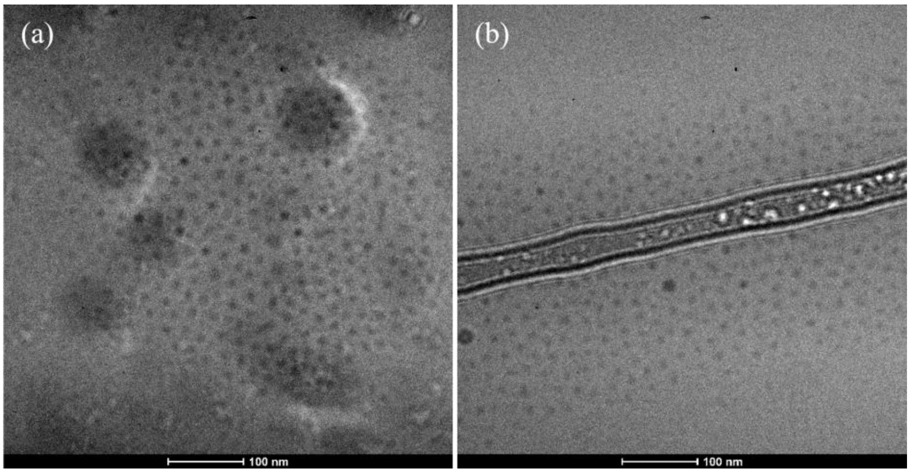


**Figure S16.** Representative cryo-TEM images of $A_{100}M_{101}D_{10}$ in aqueous solutions (1 wt.%) vitrified from (a) 20 °C ($T < T_{gel}$) and (b) 40 °C ($T > T_{gel}$), respectively.

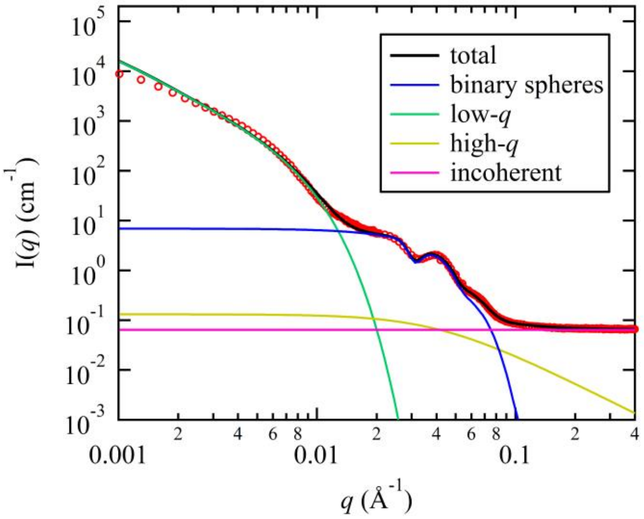


**Figure S17.** SANS profiles of 1 wt.% $A_{100}M_{101}D_{10}$ in $D_2O$ measured at 60 °C. The black line corresponds to the best fit of the data using eq. 2 along with the parameters listed in Table 2 in the main text. The colored lines represent the individual scattering contributions corresponding to different component of eq. 2: the binary sticky hard-sphere model contributions from A and D domains (the first three terms), the low-*q* scattering term, the high-*q* scattering term, and the *q*-independent incoherent background.

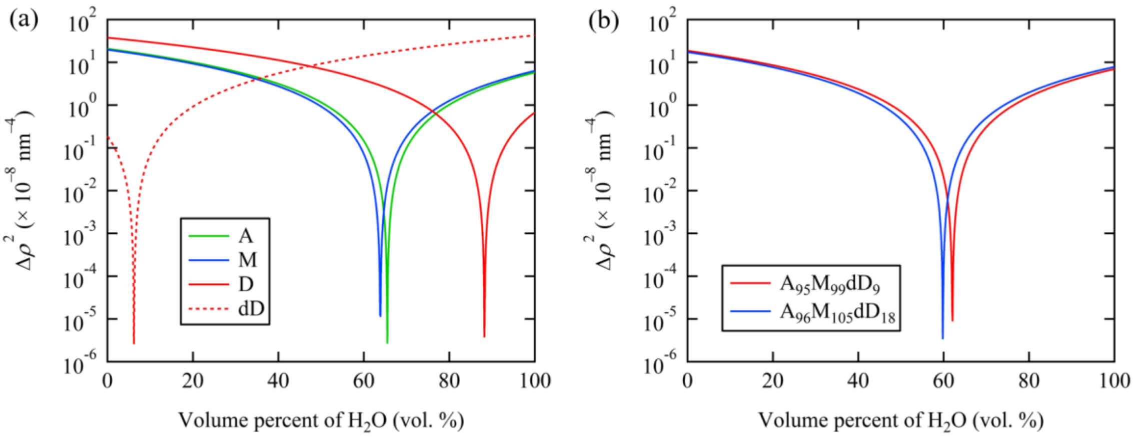


**Figure S18.** Dependence of contrast factor $\Delta\rho^2$ on the volume percent of $H_2O$ of the $H_2O/D_2O$ mixture for (a) the A, M, D, and dD individual blocks and (b) the AMdD polymers. The contrast factor $\Delta\rho^2$ is defined as $\Delta\rho^2 = (\rho_p - \rho_s)^2$, where $\rho_p$ represents the SLD of an individual block or the averaged SLD of the entire triblock terpolymer, and $\rho_s$ is the SLD of $H_2O/D_2O$ mixture. Consequently, the scattering signal from an individual block or the entire polymer, which is proportional to $\Delta\rho^2$, is minimized when $\rho_p \approx \rho_s$, i.e., $\Delta\rho^2 \approx 0$. The dip in the curve indicates the condition where the SLD of the solvent is made equal to that of an individual block, or the averaged SLD of the entire polymer. Hence, two different contrast matching conditions can be achieved by adjusting the $D_2O/H_2O$ ratio: the "dD contrast matched condition", where the dD block is contrast matched out by the solvent, and the "zero mean contrast condition", where the entire AMdD is contrast matched out by the solvent.

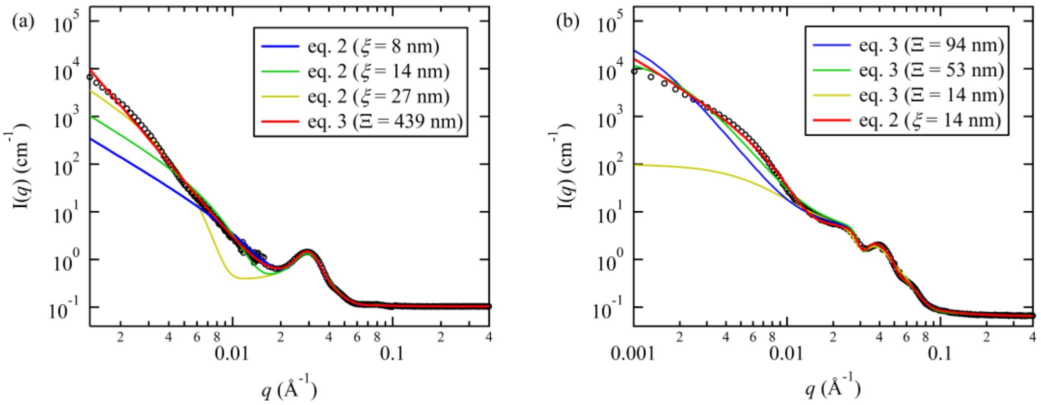


**Figure S19.** SANS profiles and the corresponding fitting curves for (a) 1 wt.% $A_{95}M_{99}dD_9$ in $D_2O/H_2O$ with 93.8 vol.% $D_2O$ (dD contrast matched condition) and (b) 1 wt.% $A_{100}M_{101}D_{10}$ in $D_2O$ at 60 °C. The open black circles represent experimental data and the colored lines represent model fittings using eq. 2 or eq. 3 with varied $\xi$ and $\Xi$ values.

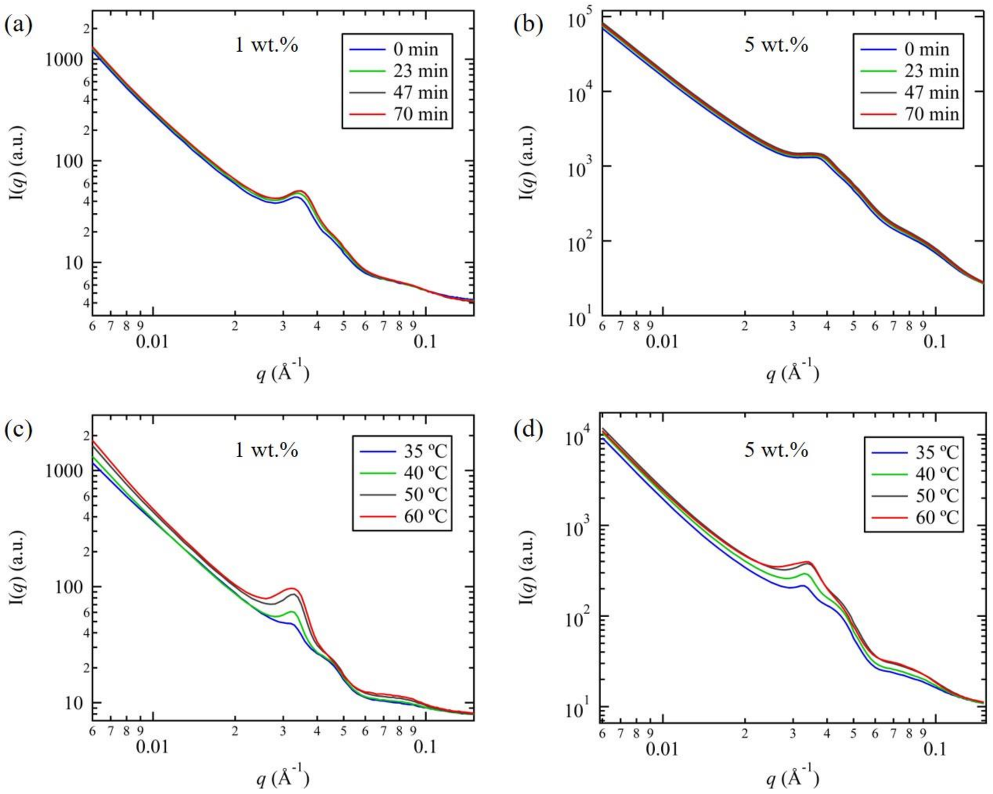


**Figure S20.** SAXS profiles of the (a) 1 wt.% and (b) 5 wt.% $A_{100}M_{101}D_{10}$ hydrogels measured at different waiting times after being heated to 40 °C. SAXS profiles of the (c) 1 wt.% and (d) 5 wt.% $A_{100}M_{101}D_{10}$ hydrogels measured at different temperatures during the heating process. For the temperature-dependent measurements, samples were equilibrated for 30 min before data acquisition. All SAXS profiles exhibit a primary peak near $q$ = 0.035 Å$^{-1}$, which is distinct from the primary peak position observed in the SANS profiles for $A_{100}M_{101}D_{10}$ in $D_2O$ (Figure 2a in the main text), while shows a closer position to that observed for $A_{95}M_{99}dD_9$ in $D_2O/H_2O$ under the dD contrast matched condition (Figure 2b in the main text). The discrepancy can be attributed to distinct interactions of X-rays and neutrons with matter, resulting in variations in contrast for different regions of the hydrogel sample. The SLD values ($\rho_{\text{x-ray}}$) for the A, M and D blocks under X-ray were estimated to be 8.8 × 10$^{-4}$, 12.8 × 10$^{-4}$ and 9.0 × 10$^{-4}$ nm$^{-2}$, respectively, using the equation $\rho_{\text{x-ray}} = n_e N_A \rho_{\text{mass}} r_e / M$, where $n_e$ is the number of electrons per block, $N_A$ is Avogadro's number, $\rho_{\text{mass}}$ is the mass density of the polymer, $r_e$ = 2.82 × 10$^{-15}$ m is classical electron radius, and $M$ is the molar mass of the block. The SLD of water under X-ray is 9.44 × 10$^{-4}$ nm$^{-2}$. As seen from the above plots, the changes in the scattering profiles are primarily temperature-dependent rather than time-dependent under isothermal condition.

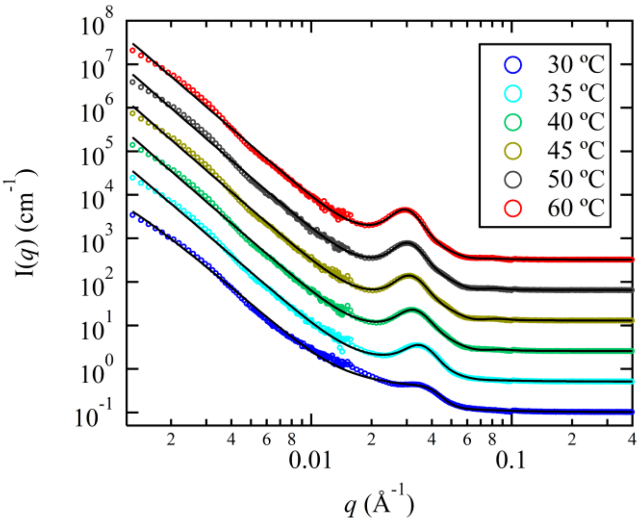


**Figure S21.** SANS profiles of 1 wt.% $A_{95}M_{99}dD_{9}$ in $D_2O/H_2O$ with 93.8 vol.% $D_2O$ (dD contrast matched condition) measured during heating process at $T \gtrsim T_{gel}$. The solid black curves correspond to the best fits of the SANS based on eq. 3 described in the main text. All profiles were shifted vertically for clarity by multiplying a factor of 5.

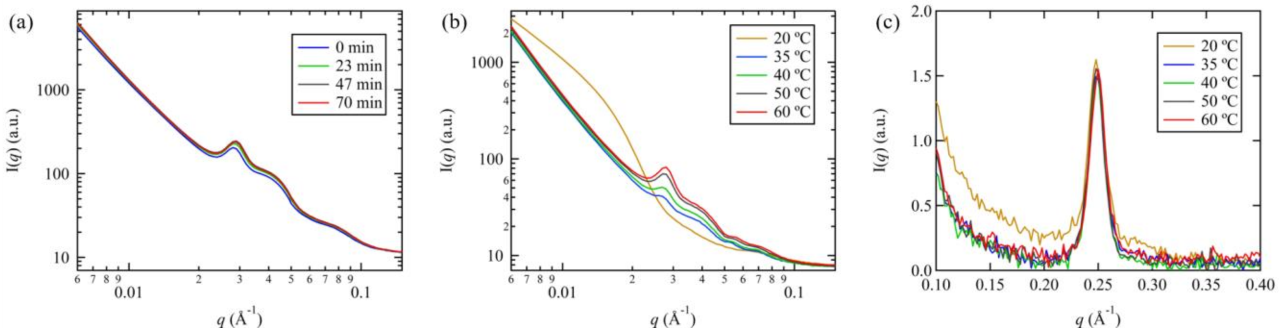


**Figure S22.** (a) SAXS profiles of the 1 wt.% $A_{101}M_{107}D_{21}$ hydrogels measured at different waiting times after being heated to 40 °C. (b) SAXS profiles of the 1 wt.% $A_{101}M_{107}D_{21}$ hydrogels measured at different temperatures during the heating process. (c) WAXS profiles of the 1 wt.% $A_{101}M_{107}D_{21}$ hydrogels near $q$ = 0.25 Å$^{-1}$ measured at different temperatures during the heating process. The scattering contribution from the pure water was subtracted from all the WAXS profiles. For the temperature-dependent measurements, samples were equilibrated for 30 min before data acquisition.

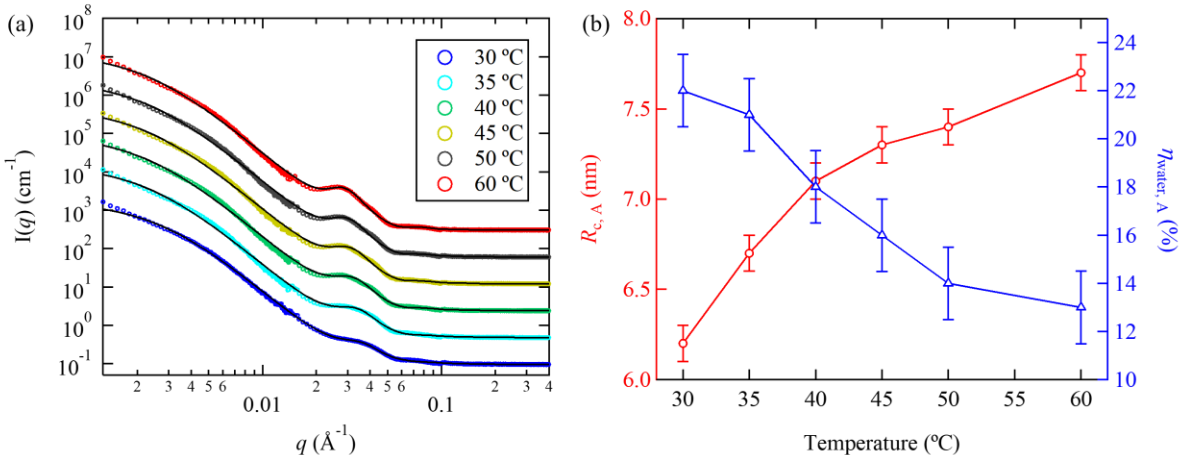


**Figure S23.** (a) SANS profiles of 1 wt.% $A_{96}M_{105}dD_{18}$ in $D_2O/H_2O$ with 93.8 vol.% $D_2O$ (dD contrast matched condition) measured during heating process at $T \gtrsim T_{gel}$. The solid black curves correspond to the best fits of the SANS based on eq. 3 described in the main text. All profiles were shifted vertically for clarity by multiplying a factor of 5. (b) Changes of core radius ($R_{c,A}$) and fraction of water content ($\eta_{water,A}$) of A domain in $A_{96}M_{105}dD_{18}$ as a function of temperature.

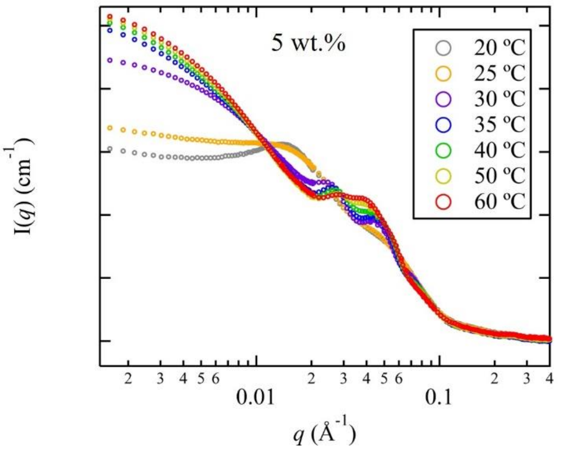


**Figure S24.** SANS profiles of 5 wt.% $A_{43}M_{92}D_9$ in $D_2O$ measured during heating process. As compared to the SANS profiles for 1 wt.% $A_{43}M_{92}D_9$ (Figure 5 in the main text), the transition point to hydrogel network is clearly decreased to lower temperature at higher polymer concentration, evidenced by the abrupt change in the SANS profile from 25 to 30 °C. At $T < T_{gel}$, the SANS profiles exhibit a pronounced scattering shoulder near $q$ = 0.015 $Å^{-1}$ due to strong intermicellar interactions. Nevertheless, the SANS profiles at $T \gtrsim T_{gel}$ display the characteristic scattering peaks with nearly identical peak positions and similar temperature dependent structural evolution to those observed at lower concentration.

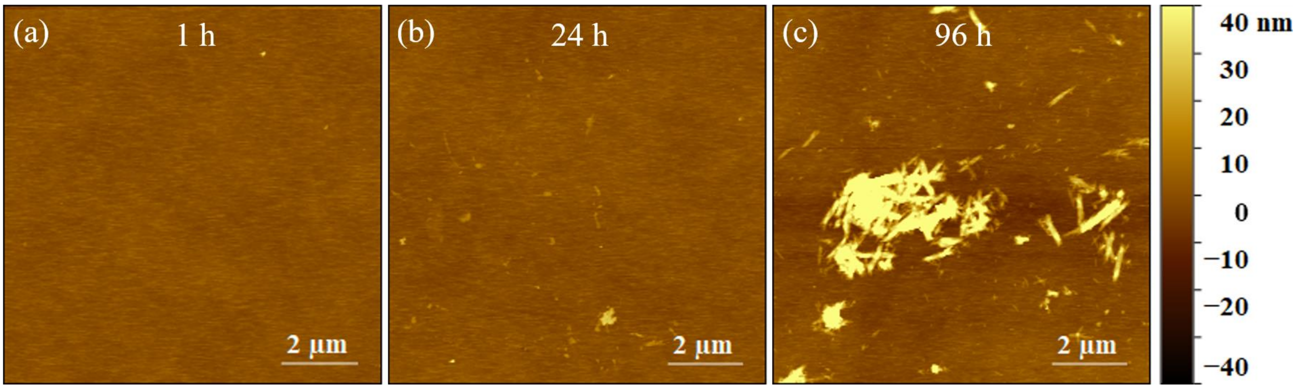


**Figure S25.** Representative atomic force microscopy (AFM) height images of the $A_{153}M_{127}D_{10}$ self-assemblies formed after aging the 1 wt.% aqueous solution at 20 °C for (a) 1 h, (b) 24 h, and (c) 96 h, under stirring at 300 rpm. For AFM sample preparation, the 1 wt.% solution was diluted for ten times and then immediately deposited onto cleaned silicon substrates via spin-coating. AFM measurements were performed using a Dimension Icon AFM (Bruker, Hamburg, Germany) in peak-force tapping mode (*i.e.*, ScanAsyst mode) under ambient conditions, using a ScanAsyst-air probe with a frequency of ~ 70 kHz and a spring constant of 0.4 N/m. The images were captured with a scan rate of ~ 1 Hz and a scanning density of 256 lines per frame.

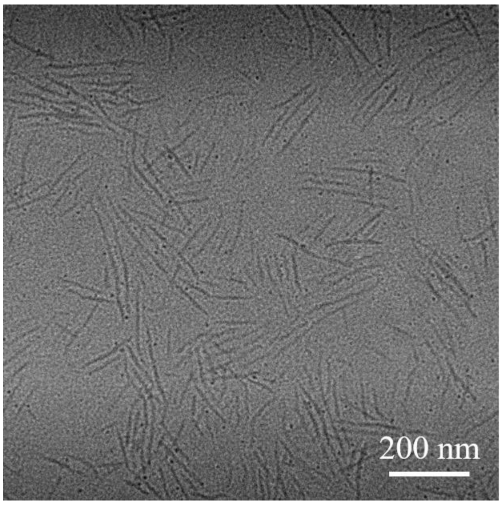


**Figure S26.** Representative cryo-TEM image of 1 wt.% $A_{153}M_{127}D_{10}$ in aqueous solution vitrified from 20 °C after the solution been aged at 20 °C by stirring under 300 rpm for about a week.

**References**


S1. Mitsunobu, O.; Wada, M.; Sano, T. Stereospecific and stereoselective reactions. I. Preparation of amines from alcohols. *J. Am. Chem. Soc.* **1972,** 94, (2), 679-680.

S2. Duncan, N. C.; Roach, B. D.; Williams, N. J.; Bonnesen, P. V.; Rajbanshi, A.; Moyer, B. A. N, n′ -dicyclohexyl-n″ -isotridecylguanidine as suppressor for the next generation caustic side solvent extraction (ng-cssx) process. *Sep. Sci. Technol.* **2012,** 47, (14-15), 2074-2087.

S3. Pedersen, J. S.; Gerstenberg, M. C. Scattering form factor of block copolymer micelles. *Macromolecules* **1996,** 29, (4), 1363-1365.

S4. Pedersen, J. S. Form factors of block copolymer micelles with spherical, ellipsoidal and cylindrical cores. *J. Appl. Crystallogr.* **2000,** 33, (3 ‐ 1), 637-640.

S5. Lau, K. H. A.; Ren, C.; Sileika, T. S.; Park, S. H.; Szleifer, I.; Messersmith, P. B. Surface-grafted polysarcosine as a peptoid antifouling polymer brush. *Langmuir* **2012,** 28, (46), 16099-16107.

S6. Sun, J.; Teran, A. A.; Liao, X. X.; Balsara, N. P.; Zuckermann, R. N. Crystallization in sequence-defined peptoid diblock copolymers induced by microphase separation. *J. Am. Chem. Soc.* **2014,** 136, (5), 2070-2077.

S7. Sun, J.; Jiang, X.; Lund, R.; Downing, K. H.; Balsara, N. P.; Zuckermann, R. N. Self-assembly of crystalline nanotubes from monodisperse amphiphilic diblock copolypeptoid tiles. *Proc. Natl. Acad. Sci. U.S.A.* **2016,** 113, (15), 3954-3959.

S8. Jiang, N.; Yu, T.; Darvish, O. A.; Qian, S.; Mkam Tsengam, I. K.; John, V.; Zhang, D. Crystallization-driven self-assembly of coil–comb-shaped polypeptoid block copolymers: Solution morphology and self-assembly pathways. *Macromolecules* **2019,** 52, (22), 8867-8877.

S9. Debye, P. Molecular-weight determination by light scattering. *J. Phys. Colloid Chem.* **1947,** 51, (1), 18-32.

S10. Pedersen, J. S. Structure factors effects in small-angle scattering from block copolymer micelles and star polymers. *J. Chem. Phys.* **2001,** 114, (6), 2839-2846.

S11. Pedersen, J. S.; Svaneborg, C.; Almdal, K.; Hamley, I. W.; Young, R. N. A small-angle neutron and x-ray contrast variation scattering study of the structure of block copolymer micelles: Corona shape and excluded volume interactions. *Macromolecules* **2003,** 36, (2), 416-433.

S12. Kinning, D. J.; Thomas, E. L. Hard-sphere interactions between spherical domains in diblock copolymers. *Macromolecules* **1984,** 17, (9), 1712-1718.

S13. Zhou, C.; Toombes, G. E. S.; Wasbrough, M. J.; Hillmyer, M. A.; Lodge, T. P. Structure of two-compartment hydrogels from thermoresponsive abc triblock terpolymers. *Macromolecules* **2015,** 48, (16), 5934-5943.

S14. Pedersen, J. S.; Hamley, I. W.; Ryu, C. Y.; Lodge, T. P. Contrast variation small-angle neutron scattering study of the structure of block copolymer micelles in a slightly selective solvent at semidilute concentrations. *Macromolecules* **2000,** 33, (2), 542-550.

S15. Baxter, R. J. Percus–yevick equation for hard spheres with surface adhesion. *J. Chem. Phys.* **1968,** 49, (6), 2770-2774.

S16. Menon, S. V. G.; Manohar, C.; Rao, K. S. A new interpretation of the sticky hard sphere model. *J. Chem. Phys.* **1991,** 95, (12), 9186-9190.

S17. Kotlarchyk, M.; Chen, S. H. Analysis of small angle neutron scattering spectra from polydisperse interacting colloids. *J. Chem. Phys.* **1983,** 79, (5), 2461-2469.
